# Structure activity relationships and the binding mode of quinolinone-pyrimidine hybrids as reversal agents of multidrug resistance mediated by P-gp

**DOI:** 10.1038/s41598-021-96226-6

**Published:** 2021-08-19

**Authors:** Jerónimo Laiolo, Priscila Ailin Lanza, Oscar Parravicini, Cecilia Barbieri, Daniel Insuasty, Justo Cobo, D. Mariano Adolfo Vera, Ricardo Daniel Enriz, Maria Cecilia Carpinella

**Affiliations:** 1 Fine Chemical and Natural Products Laboratory, IRNASUS CONICET-UCC, Universidad Católica de Córdoba, Avda. Armada Argentina 3555, X5016DHK Córdoba, Argentina; 2 Department of Chemistry, College of Exact and Natural Sciences, Universidad Nacional de Mar del Plata - QUIAMM – INBIOTEC CONICET, Funes 3350, Mar del Plata, Argentina; 3 Faculty of Chemistry, Biochemistry and Pharmacy, Universidad Nacional de San Luis, IMIBIO-SL, Ejército de los Andes 950, 5700 San Luis, Argentina; 4 Department of Chemistry and Biology, Universidad del Norte, Km 5 vía Puerto Colombia, 081007 Barranquilla, Colombia; 5 Department of Inorganic and Organic Chemistry, Universidad de Jaén, Campus Las Lagunillas s/n, 23071 Jaén, Spain

**Keywords:** Drug discovery, Chemistry, Mathematics and computing

## Abstract

P-gp-associated multidrug resistance is a major impediment to the success of chemotherapy. With the aim of finding non-toxic and effective P-gp inhibitors, we investigated a panel of quinolin-2-one-pyrimidine hybrids. Among the active compounds, two of them significantly increased intracellular doxorubicin and rhodamine 123 accumulation by inhibiting the efflux mediated by P-gp and restored doxorubicin toxicity at nanomolar range. Structure–activity relationships showed that the number of methoxy groups, an optimal length of the molecule in its extended conformation, and at least one flexible methylene group bridging the quinolinone to the moiety bearing the pyrimidine favored the inhibitory potency of P-gp. The best compounds showed a similar binding pattern and interactions to those of doxorubicin and tariquidar, as revealed by MD and hybrid QM/MM simulations performed with the recent experimental structure of P-gp co-crystallized with paclitaxel. Analysis of the molecular interactions stabilizing the different molecular complexes determined by MD and QTAIM showed that binding to key residues from TMH 4–7 and 12 is required for inhibition.

## Introduction

Cancer is a complex disease which is among the major causes of death worldwide^1^. Chemotherapy plays a key role among the strategies to treat cancer^2^ in particular for the treatment of hematological malignancies^3^. Despite its therapeutic advantages, the success of this treatment is impeded by the development of resistance to conventional chemotherapeutic drugs^4^, which is a major negative factor for the survival of patients^2^. One primary mechanism involved in this phenomenon is the over-expression of P-glycoprotein (P-gp), an ATP binding cassette (ABC) transmembrane protein that pumps a great variety of anticancer drugs out from the cell^5^. This efflux leads to a diminished intracellular concentration and a consequent insensitivity, which is known as multidrug resistance (MDR)^6^. The increased expression of P-gp is decisive in the resistance of different types of cancer^7^. In chronic myelogenous leukemia more than a half of the patients were unresponsive to Vinca alkaloids and anthracyclines due to its presence^8^. Structurally, P-gp consists of two homologous transmembrane domains (TMD), each formed by six transmembrane α-helices (TMHs) and two cytosolic nucleotide-binding domains (NBDs)^9^.

Considerable attempts have been made to find P-gp-interacting compounds, but clinical trials with three generations of modulators have failed for diverse reasons^5,10^, among these, side effects, lack of selectivity, low efficacy and, most important, failures in the design of the trials^11,12^. It is thus a matter of great concern to develop novel chemical entities able to tackle P-gp-mediated outward transport and thus overcome MDR in cancer cells. In addition, efforts should be made to establish the chemical groups able to interact with certain amino acids present in TMHs that allow a more complete understanding about human P-gp inhibition.

The importance of the 6,7-dimethoxy-1,2,3,4-tetrahydroisoquinoline moiety in the highly effective third-generation P-gp inhibitors, tariquidar, which also bears an isoquinoline group, elacridar, HM30181, WK-X-34^13,14^ and their derivatives, some containing aromatic amide^2,14^, as well as the presence of an aminopyrimidine moiety in the P-gp inhibitor, imatinib^15^, and the promising MDR reversal activity of the multi-substituted or fused pyrimidine scaffold^16^, encouraged us to search for potential P-gp inhibitors in a library of novel quinolin-2-one-pyrimidine hybrids^17^. Previous reports showed different series of compounds bearing the tetrahydroisoquinoline scaffold with significant activity, at nanomolar range, for reversing P-gp-mediated drug resistance^18–22^.

The present work investigated the ability of a panel of quinolin-2-one-pyrimidine hybrids to inhibit P-gp functionality and to restore sensitivity to the chemotherapeutic drug, doxorubicin (Dox), in multi-resistant Lucena 1 leukemia cells. The various interactions that accommodate ligands to certain sites of P-gp could be explained in depth by understanding the stereo-electronic intricacies involved in their binding modes. With this aim, the possible molecular complexes were evaluated using combined molecular modeling techniques.

## Results and discussion

### Classes of tested compounds

**Figure 1:**
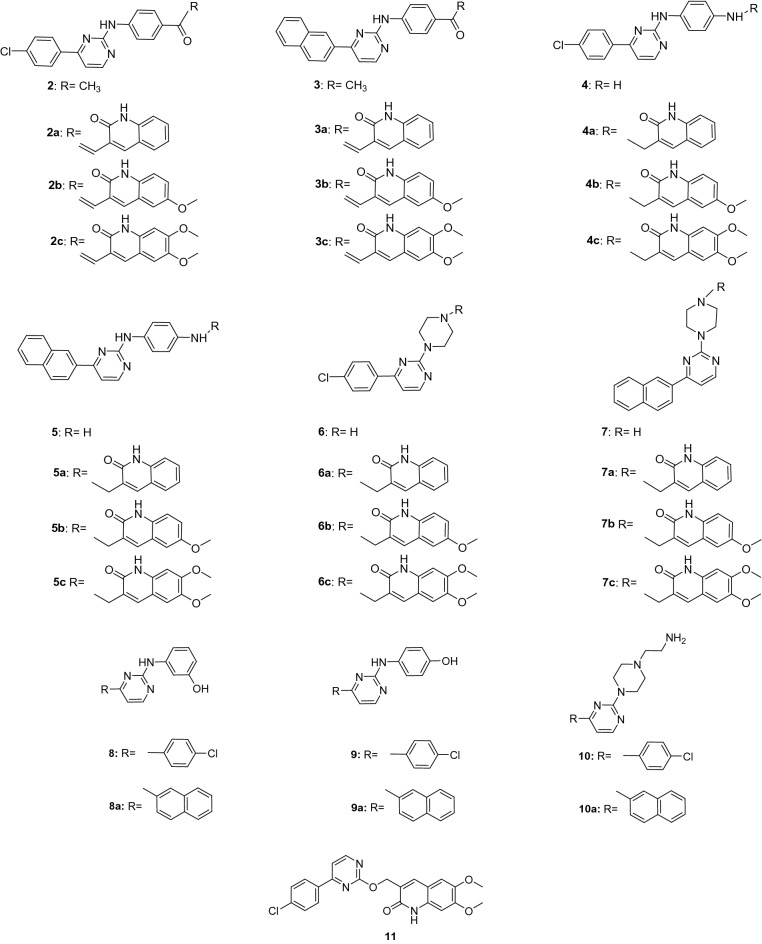
Chemical structures and labelling of the tested compounds.

All compounds depicted in Fig. 1 were prepared as previously reported^17^. The synthesis of 2-oxoquinoline-3-carbaldehydes (**Ia–c**) (Supplementary Fig. S1, online) started with the building of the quinoline core via the Meth–Cohn method^17,23^ from the corresponding acetanilides by treatment under Vielsmeier’s conditions, which gave rise to 2-chloroquinoline-3-carbaldehyde derivatives. These were transformed into the corresponding quinolone derivatives (**Ia–c**) by aqueous acetic acid hydrolysis, and were then reduced with sodium borohydride to 3-(hydroxymethyl)quinolinones (**IIa–c**), which with thionyl chloride afforded the 3-(chloromethyl)quinolinones (**IIIa–c**) (Supplementary Fig. S1, online). The starting material 2-chloro-4-(4-aryl)pyrimidines **(1a–b)** (**a** for 4-chlorophenyl and **b** for naphtha-2-yl) were prepared by Suzuki reaction from commercial 2,4-dichloropyrimidine with the corresponding arylboronic acid^24,25^ and used as precursors to give the pyrimidine derivatives **2–11** by aromatic nucleophilic substitution (see Supplementary Fig. S2, for reaction conditions, online). Tthe amino residue linked to the pyrimidine ring at position C-2 were, respectively, *p*-aminoacetophenone (**2**, **3**), *p*-phenylenediamine (**4**, **5**), piperazine (**6**, **7**), *m*-aminophenol (**8**, **8a**), *p*-aminophenol (**9**, **9a**), 2-(piperazin-1-yl)ethan-1-amine (**10**, **10**a). Finally, the quinolinone residue was coupled to derivatives **2**, **3**, **4**, **5**, **6** and **7**, as summarized in Supplementary Fig. S2, online, to give the corresponding derivatives **2a–c**, **3a–c**, **4a–c**, **5a–c**, **6a–c** and **7a–c**. The reaction of pyrimidine **1a** with **IIc** afforded compound **11** in which pyrimidine and dimethoxyquinolon-2-ylmethyloxy moieties were linked without any spacer between them.

### Inhibitory effects on P-gp-mediated outward transport by the target compounds, cytotoxicity and SAR studies

**Figure 2:**
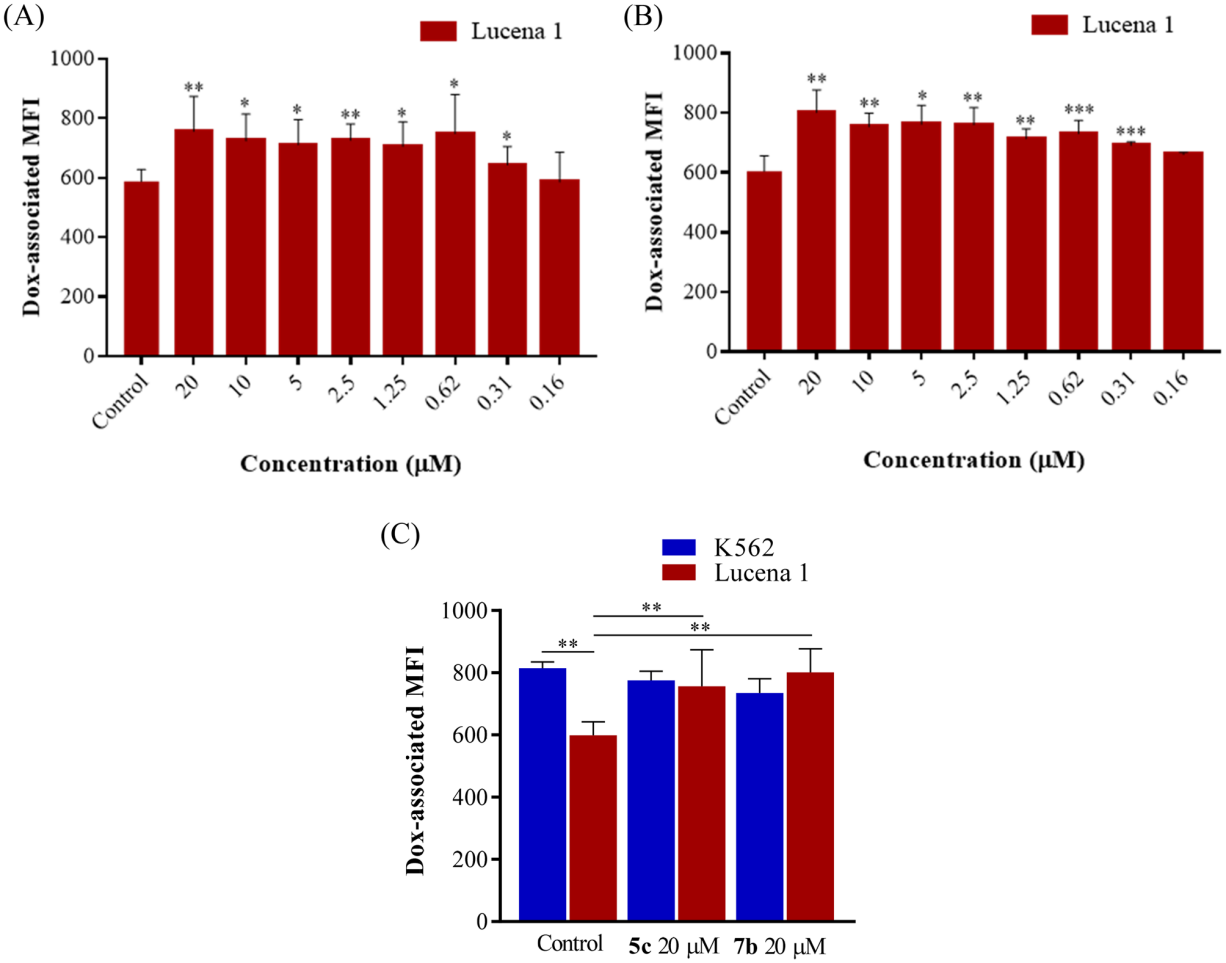
Inhibition on P-gp doxorubicin (Dox) outward transport by different concentrations of compounds **5c** (**A**) and **7b** (**B**), determined by accumulation assay in Lucena 1 and (**C**) at 20 μM of both compounds in Lucena 1 and K562 cells. Doxorubicin-associated intracellular fluorescence significantly increased in Lucena 1 by treatments with selected compounds but not in K562. Significant differences relative to the respective negative control were determined by using a paired one-tailed Student’s t test (***p < 0.001, **p < 0.01, *p < 0.05).

Flow cytometry findings showed that compounds **2**, **2a–c**, **3a–c**, **4**, **5**, **8**, **8a**, **9** and **9a** (Fig. 1), tested at 20 µM, were ineffective (*p* > 0.05) for increasing the intracellular accumulation of the fluorescent substrate Dox. However, compounds **3**, **4a–c**, **5a–c**, **6**, **6a–c**, **7**, **7a–c**, **10**, **10a** and **11** (Fig. 1) at 20 µM significantly enhanced Dox retention (*p* > 0.001–0.05) with fluorescence intensity ratio (FIR) values ranging from 1.21 to 2.87, by efficiently blocking the efflux function of P-gp (Table 1). Therefore, these compounds were further evaluated at serial dilutions. As the results show in Table 1, compounds **7a**, **10**, **10a** and **11** were able to induce a higher accumulation of Dox than the negative control, as from 1.25 µM (*p* < 0.05) followed by compounds **4c**, **5b**, **6c** and **7c** which showed minimum effective concentrations (MECs) of 0.62 µM (*p* < 0.05). Compounds **5c** and **7b,** were the most potent, as they still exhibited effectiveness at 0.31 µM (*p* < 0.05 and 0.001, respectively) (Table 1, Fig. 2A,B). The efficacy of compounds **5c** and **7b** was similar to that observed with the classical P-gp inhibitor, verapamil, at all the concentrations assayed (*p* > 0.05), except at 0.31 µM (*p* < 0.05 and 0.01, respectively). The intracellular fluorescence-associated values in Lucena 1 cells treated with both compounds at 20 µM were similar to those observed in treated and untreated sensitive K562 cells (*p* > 0.05), which in turn showed no differences in their intracellular Dox fluorescence (*p* > 0.05) (Fig. 2C). These results suggested a complete reversal effect on P-gp-mediated MDR and a selective inhibition on P-gp function.

**Table 1:** Inhibitory effect of target compounds on P-gp transport activity in Lucena 1 and K562 leukemia cells.

Compound	FIR Lucena 1										FIR K562		
	Concentration (µM)										Concentration (µM)		
	20	10	5	2.50	1.25	0.62	0.31	0.16	0.08	0.04	20	10	5
**4a**	1.21 ± 0.04***	1.03 ± 0.01									0.89 ± 0.03*		
**3**	1.70 ± 0.22**	1.24 ± 0.02*	1.06 ± 0.05								0.98 ± 0.07		
**6a**	1.40 ± 0.05***	1.30 ± 0.05*	1.15 ± 0.05*	1.06 ± 0.04							0.92 ± 0.04		
**7**	2.23 ± 0.26***	1.42 ± 0.02*	1.19 ± 0.05*	1.07 ± 0.10							1.20 ± 0.06*	1.10 ± 0.08	
**4b**	1.29 ± 0.08**	1.23 ± 0.08*	1.20 ± 0.06*	1.20 ± 0.06*	1.10 ± 0.13						0.90 ± 0.03**		
**5a**	1.24 ± 0.07**	1.10 ± 0.02*	1.06 ± 0.01*	1.05 ± 0.01**	0.95 ± 0.05						0.86 ± 0.02*		
**6**	1.65 ± 0.12***	1.38 ± 0.08*	1.18 ± 0.03*	1.18 ± 0.04*	1.09 ± 0.06						1.20 ± 0.02****	1.19 ± 0.02***	1.09 ± 0.04
**6b**	1.55 ± 0.07***	1.44 ± 0.13*	1.32 ± 0.09*	1.15 ± 0.05*	1.01 ± 0.03						0.94 ± 0.10		
**7a**	1.33 ± 0.11*	1.52 ± 0.17**	1.38 ± 0.11*	1.28 ± 0.12*	1.10 ± 0.03*	0.95 ± 0.02					1.02 ± 0.07		
**10**	1.72 ± 0.14**	1.30 ± 0.05*	1.41 ± 0.09*	1.26 ± 0.04**	1.11 ± 0.03*	1.06 ± 0.04					1.18 ± 0.03*	0.98 ± 0.06	
**10a**	2.36 ± 0.25***	1.63 ± 0.17**	1.30 ± 0.08**	1.21 ± 0.02**	1.13 ± 0.03*	1.04 ± 0.03					1.26 ± 0.06**	1.13 ± 0.02**	0.98 ± 0.01
**11**	2.87 ± 0.55**	1.30 ± 0.07**	1.38 ± 0.07**	1.23 ± 0.04**	1.14 ± 0.02*	1.02 ± 0.02					1.44 ± 0.03*	1.01 ± 0.02	
**4c**	1.39 ± 0.07**	1.28 ± 0.07*	1.32 ± 0.09*	1.31 ± 0.11*	1.24 ± 0.08*	1.11 ± 0.03*	1.03 ± 0.01				0.93 ± 0.02*		
**5b**	1.36 ± 0.08***	1.25 ± 0.07*	1.31 ± 0.08**	1.29 ± 0.08*	1.21 ± 0.07*	1.16 ± 0.07*	1.07 ± 0.03				0.96 ± 0.02		
**6c**	1.68 ± 0.12**	1.49 ± 0.20*	1.32 ± 0.09*	1.32 ± 0.08*	1.19 ± 0.07*	1.11 ± 0.03*	1.00 ± 0.01				0.79 ± 0.007**		
**7c**	1.45 ± 0.10***	1.29 ± 0.11*	1.27 ± 0.04*	1.23 ± 0.02**	1.24 ± 0.05*	1.22 ± 0.07*	1.08 ± 0.05				1.04 ± 0.03		
**5c**	1.42 ± 0.09**	1.24 ± 0.10*	1.23 ± 0.05*	1.26 ± 0.01**	1.22 ± 0.03*	1.29 ± 0.08*	1.12 ± 0.02*	0.99 ± 0.07			0.95 ± 0.02		
**7b**	1.43 ± 0.10**	1.25 ± 0.04**	1.26 ± 0.04*	1.26 ± 0.04**	1.18 ± 0.03**	1.21 ± 0.01***	1.10 ± 0.01***	1.04 ± 0.02			0.88 ± 0.02*		
Ver	1.55 ± 0.11****	1.38 ± 0.04****	1.34 ± 0.05***	1.31 ± 0.03***	1.29 ± 0.05***	1.22 ± 0.04**	1.23 ± 0.03***	1.14 ± 0.005***	1.11 ± 0.02*	1.09 ± 0.03	1.08 ± 0.05		

Fluorescence intensity ratio (FIR) = mean fluorescence intensity (MFI) of doxorubicin (Dox) with compound/MFI of Dox alone. Ver: verapamil. Significant differences from the negative control were determined by using a paired one-tailed Student’s t test (****p < 0.0001, ***p < 0.001, **p < 0.01, *p < 0.05).

With the exception of compounds **7c** and **11** having IC_50_ values of 6.7 ± 0.20 and 3.8 ± 0.10 µM, respectively, against K562, all of the compounds showing modulatory activity were non-cytotoxic against the sensitive cells and the resistant counterpart as is evident from the IC_50_ values obtained, which were higher than 10 µM, the threshold established by the US National Cancer Institute for considering a compound as cytotoxic^3^. The IC_50_ values for the non-toxic compounds were in all cases above 20 µM, except for compound **10a** showing an IC_50_ value of 17.7 ± 0.15 µM against K562 and compounds **7**, **7c**, **10a** and **11** with IC_50_ values of 17.9 ± 0.55, 10.2 ± 0.23, 17.1 ± 0.95 and 10.7 ± 0.15 µM, respectively against Lucena 1. Specifically, the most active compounds **5c** and **7b** were non-toxic, fulfilling the requisite of negligible cytotoxicity of the compound alone for developing effective P-gp modulators^26,27^.

Previously, it was found that compounds **2b**, **3b**–**c**, **5b**–**5c** and **11** exhibited inhibition against sphingosine kinases SphK1 and SphK2, with IC_50_ values ranging from 3.1 to 25.2 µM^17^. It has been proposed that the overexpression of SphK1 increased the level of P-gp, while SphK inhibitors down-regulated the expression of the transporter^28–30^. Although compounds **2b** and **3b**–**c** were inactive for inhibiting P-gp, they could reduce P-gp expression through their significant activity against SphK1 and SphK2. In contrast, compounds **5b**–**c** and **11** were able to do the same in addition to their anti-P-gp activity. The highly effective compound **7b** was inactive against the kinases, with an IC_50_ value > 650 µM. Similar results were obtained for compounds **4b**–**c**, **5a**, **6**, **6a**–**c**, **7a** and **7c**. The most effective compounds of this series, **6c**, 7**b**–**c**, which are hit compounds and therefore the best candidates to be taken as starting structures to carry out structural changes to obtain better ligands, showed no activity on SphK1 and SphK2.

**Figure 3:**
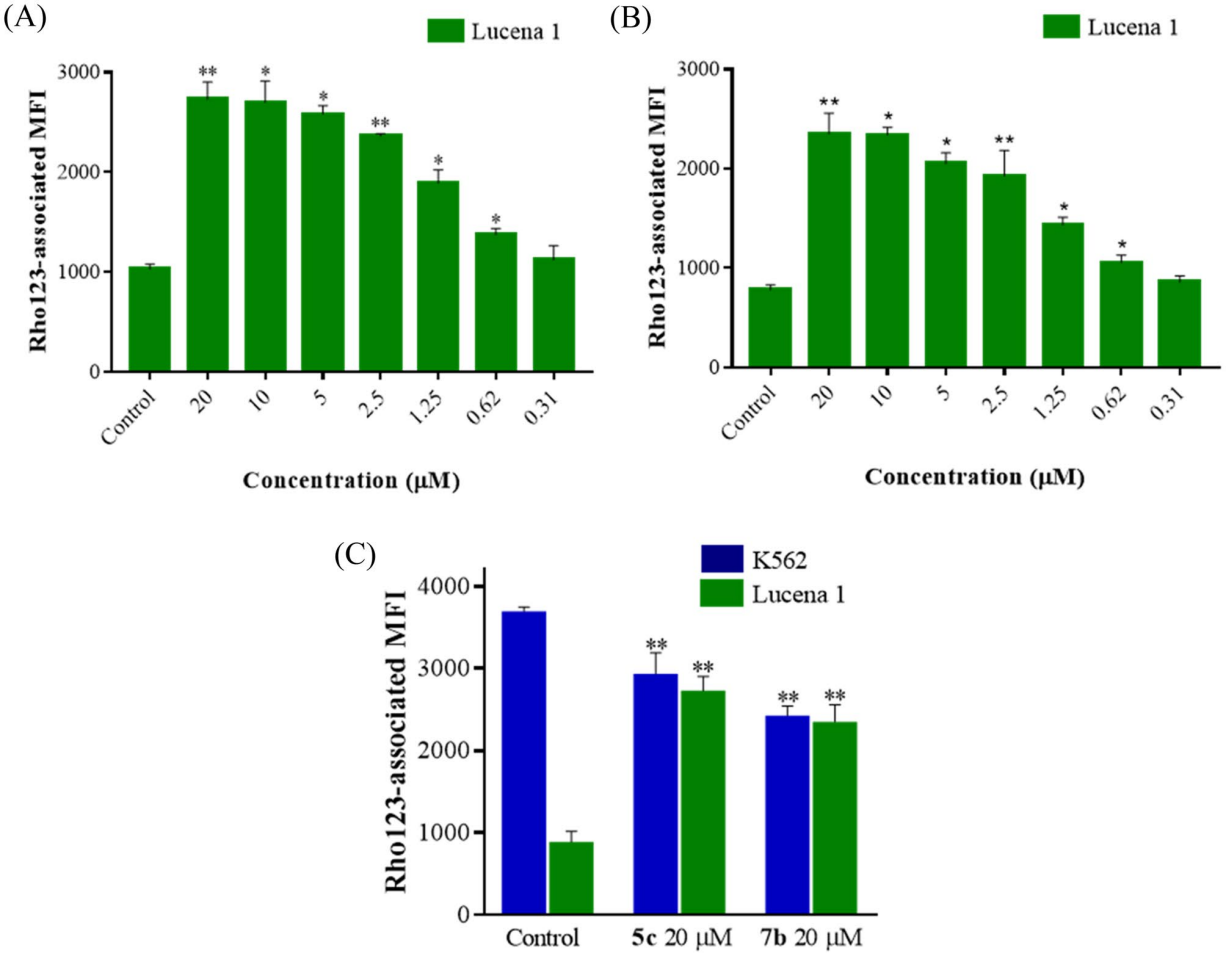
Inhibition on P-gp rhodamine 123 (Rho123) outward transport by different concentrations of compounds **5c** (**A**) and **7b** (**B**) determined by accumulation assay in Lucena 1 and (**C**) at 20 μM of both compounds in Lucena 1 and K562 cells. Rhodamine 123 associated intracellular fluorescence significantly increased in Lucena 1 by treatments with selected compounds but not in K562. Significant differences relative to the respective negative control were determined by using a paired one-tailed Student’s t test (**p < 0.01, *p < 0.05).

Rhodamine 123 (Rho123) is a fluorescent probe efficiently effluxed by P-gp, which binds to the transporter at sites distinct from those of Dox^5^. Therefore, the ability of compounds **5c** and **7b** to enhance Rho123-associated intracellular fluorescence was further explored by flow cytometry. As shown in Fig. 3A,B, Lucena 1 cells treated with compounds **5c** and **7b** at 20 µM retained 2.72 ± 0.10 and 3.05 ± 0.16 -fold more Rho123 than the untreated cells (*p* < 0.01) with MECs of 0.62 µM (FIR = 1.36 ± 0.04 and 1.37 ± 0.06, respectively, *p* < 0.05). In comparison, both compounds increased the accumulation of Rho123 with efficiency (*p* > 0.05) similar to that of verapamil at 20 and 0.62 µM (FIR = 4.40 ± 0.82 and 1.22 ± 0.04). Meanwhile, no increase in Rho123 accumulation was observed in K562 (Fig. 3C), thus showing an effect ascribed only to inhibition of the P-gp extruding function.

It has been previously described that the number of hydrogen bond acceptor methoxy groups on terminal phenyl rings is favorable for P-gp inhibitory activity^31–33^. Reflecting this, compound **5c**, bearing two OCH_3_ groups, displayed pronounced activity, followed by **5b** with one methoxy and then compound **5a** lacking this substituent (MECs = 0.31, 0.62 and 2.50 μM, respectively). The same trend was observed with compounds **7c**, **7b** and **7a** (MECs = 0.62, 0.31 and 1.25 μM, respectively) (Table 1).

It is important to highlight that although no microprecipitation was observed during the assay, the slightly higher insolubility of the dimethoxy compound, **7c**, compared to the monomethoxy compound **7b**, may have slightly masked its anti-P-gp effect, therefore showing a higher minimum effective concentration (MEC) value than expected. Increasing the number of methoxy substituents also resulted in improved activity in the chlorinated compounds **4c** and **4b** compared to compound **4a** (MECs = 0.62, 2.5 and 20 μM, respectively) (Table 1). The same was observed with the chloride compounds **6c** and **6b** with respect to compound **6a** (MECs = 0.62, 2.5 and 5 μM, respectively). The presence of the *p*-chlorophenyl group was detrimental to the inhibitory effect of compounds **4a**–**b** (MECs = 20 and 2.5 μM, respectively) and **6a**–**b** (MECs = 5 and 2.5 μM, respectively) in comparison to their respective analogues **5a**–**b** and **7a**–**b** which instead bear a naphtha-2-yl group. Interestingly, the presence of two OCH_3_ groups counteracts the adverse effect exerted by the chlorobenzene substituent, as was observed when compounds **4b** and **6b** (MECs = 2.5 μM) were compared with their respective analogues **4c** and **6c**, showing MEC values of 0.62 μM, the latter with similar activity to that of the closely related compounds **5c** and **7c**, respectively (Table 1). It has been previously reported that compounds featuring chlorine at *ortho*-, *meta*- and *para*-positions, also bearing isoquinolines with two methoxy groups, exhibited similar P-gp modulatory activity to that observed with verapamil^13^. The results obtained clearly indicated that the methoxy substituents significantly influenced P-gp inhibition. The replacement of the *p*-phenylenediamine in compounds **4a–c** and **5a–c** for a carbonyl group as in the respective compounds **2a–c** and **3a–c** prevented the anti-P-gp effect, regardless of the presence of OCH_3_ or chloride substituents (Table 1). The clear loss of activity of these compounds was further revealed by docking and molecular dynamics (MD) simulations.

### Reversal activity on doxorubicin toxicity of the most promising compounds 5c and 7b

**Figure 4:**
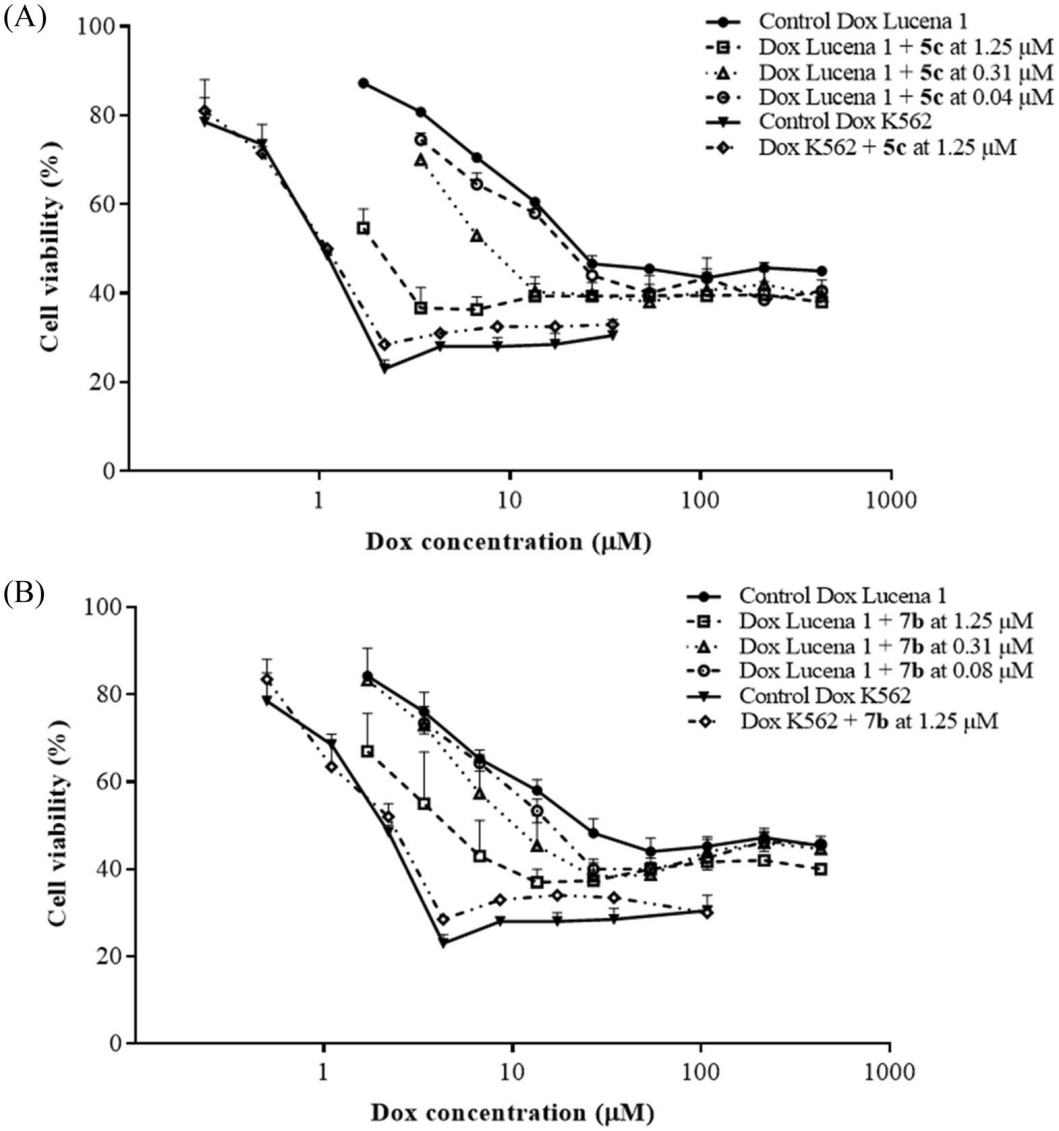
Dose–response curves for restoring sensitivity in Lucena 1 treated with doxorubicin (Dox) as an antineoplastic drug in the absence or presence of (**A**) compound **5c** and (**B**) compound **7b**. In Lucena 1 cells, doxorubicin toxicity was significantly increased when compounds **5c** and **7b** were co-administered from 0.04 and 0.08 µM, respectively (*p* < 0.05). The same assay was performed in K562 to discard additional effects other than P-gp. Values are expressed as means ± SE of at least three independent experiments.

To validate the assumption that increased accumulation of Dox is associated with a concomitant enhancement in its cytotoxicity, compounds **5c** and **7b** were co-administered with this chemotherapeutic drug and the effect on cell viability was determined. Consistent with the accumulation of Dox, compounds **5c** and **7b** at 1.25 µM substantially decreased the half-maximal inhibitory concentration (IC_50_) values of Dox (Fig. 4), circumventing Lucena 1 resistance with fold reversal (FR) values of 5.16 ± 0.60 and 6.90 ± 2.84, respectively, and showing a similar potency to that of verapamil (*p* > 0.05) with a FR value of 6.21 ± 1.28. Compound **5c** was still able to reverse Dox resistance when the concentration decreased to 40 nM (FR = 1.30 ± 0.05), while compound **7b** still chemosensitized Lucena 1 at 80 nM (FR = 1.51 ± 0.23) (Fig. 4). The activity observed with **5c** and **7b** was not significantly different (*p* > 0.05) than that of verapamil (FR = 1.55 ± 0.17 and 1.40 ± 0.11 at 40 and 80 nM, respectively).

### Molecular modelling

With the aim of gaining insight into the way that ligands interact with P-gp, molecular modeling was performed. The protocol for this analysis was divided into three interrelated parts with increasing levels of detail. First, in the docking protocol, the interior of the whole transmembrane region was scanned, making no assumptions about the localization of a particular binding site. The main binding sites were determined for the experimental reference chemotherapeutic drug, Dox, the known reference inhibitors tariquidar and verapamil and the subject compounds shown in Fig. 1. Next, detailed analyses were performed of the binding dynamics of the selected species with poor, mild or high activity, and an estimate of each free energy of binding was calculated by means of classical MD simulations. The trajectories obtained were also analyzed to identify the main contributions to these by each residue. Finally, each interaction was discussed in more detail in the light of the QTAIM analyses of hybrid QM/MM calculations.

**Figure 5:**
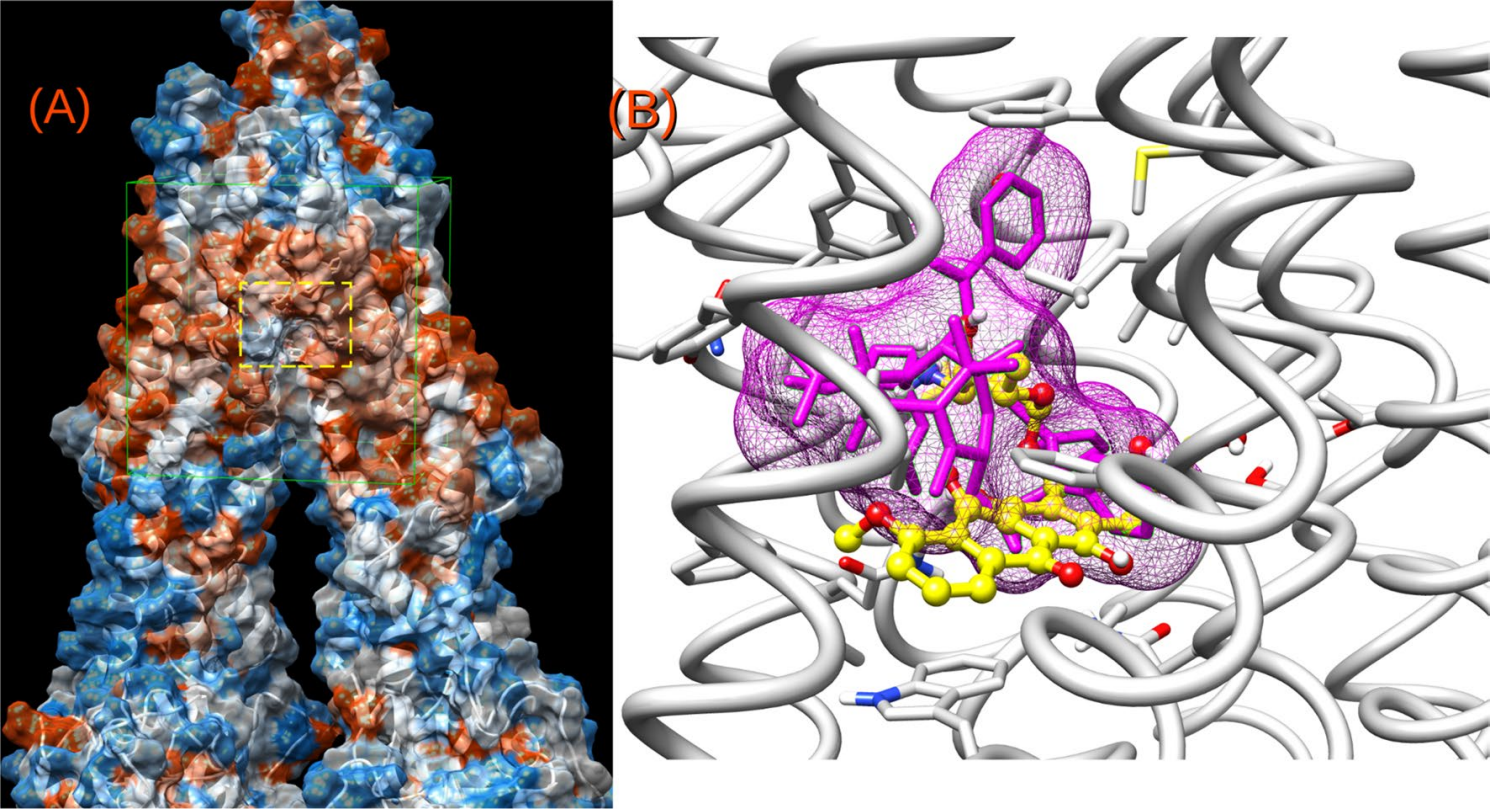
(**A**) Distant view of the P-gp surrounded by a molecular surface (probe 1.4 Å) colored according to its hydrophobicity (orange → most hydrophobic). The green cube indicates the docking region; the dotted yellow rectangle is zoomed in (**B**). (**B**) Superimposition of doxorubicin (yellow) and the co-crystallized chemotherapeutic drug, Taxol (magenta).

**Figure 6:**
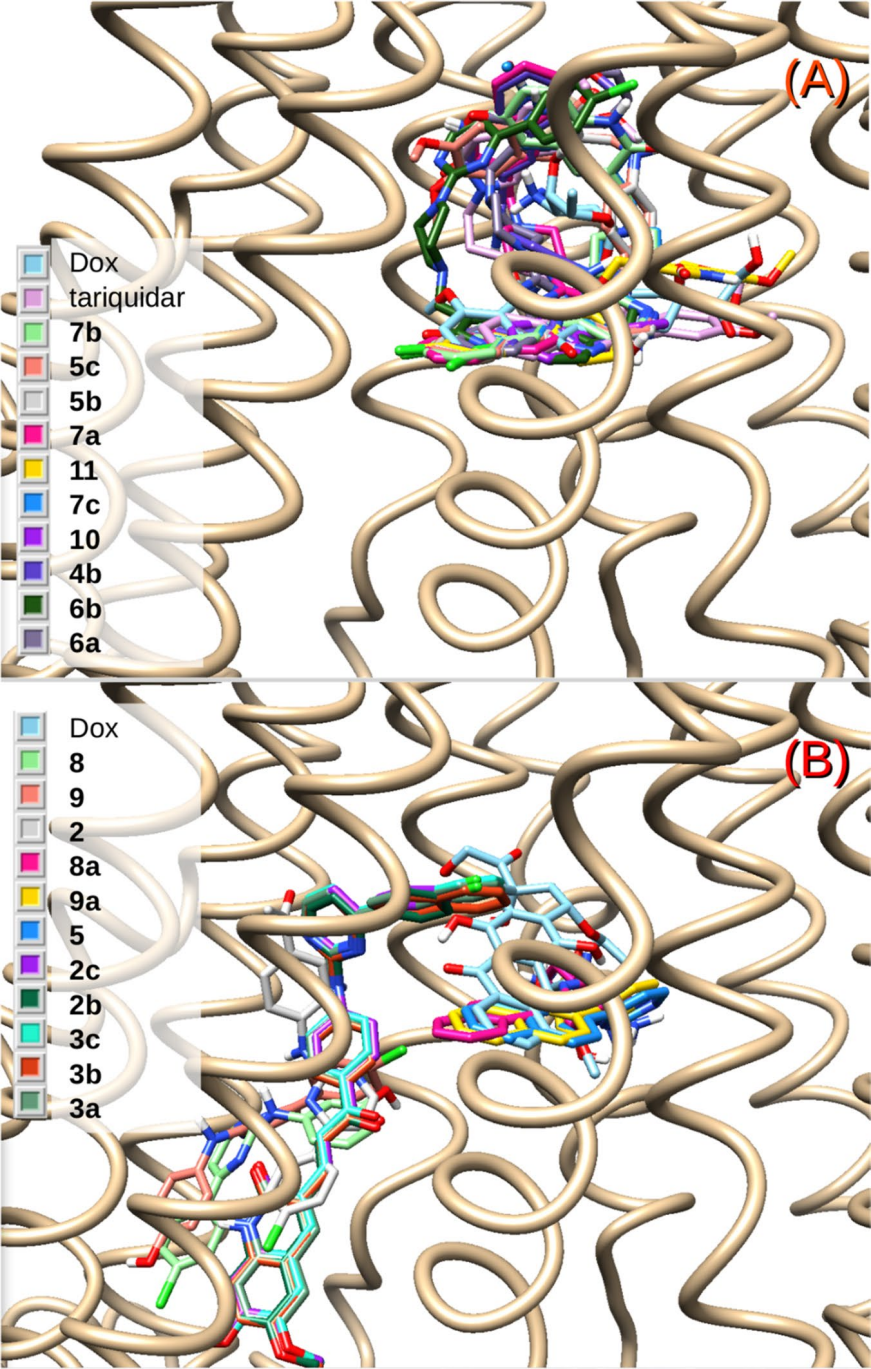
(**A**) Superimposition of the docked structures of the active compounds **4b**, **5b–c**, **6a–b**, **7a–c**, **10** and **11**, together with the chemotherapeutic substrate doxorubicin and tariquidar. (**B**) The same for poor or inactive compounds **2**, **2b–c**, **3a–c**, **5**, **8**, **8a**, **9** and **9a**.

The primary binding region for the experimental reference chemotherapeutic drug, Dox, was found to be overlapped with the region of the bulkier cytotoxic agent paclitaxel (Taxol), which was co-crystallized in the novel P-gp structure (Fig. 5)^34^. The docking results started to shed light on the differences in the activities of the panel of assayed compounds, even for those having similar structures but sharp differences in Dox accumulation profiles. Most of the mild and all the powerful inhibitors fell inside the site also shared by Dox and Taxol (Fig. 5), involving strong interactions with the aromatic residues, mainly from TMH 4, 5 and 6 from one homologous half and with those from TMH 7 and 12 from the other half of the transporter. Figure 6A shows a superimposition of the most stable docked poses of active compounds **4b**, **5b–c**, **6a–b**, **7a–c**, **10** and **11** along with the top reference inhibitor, tariquidar and Dox. A gross common feature for all these poses in this binding pocket is that they all fold into a distorted “U”-shape. The aromatic edges of each compound accommodated as the branches of the “U” with an aliphatic/conjugated flexible bridge as its camber.

This flexible intermediate portion, which also itself interacts with TMH 12, allowed for the turn or camber. In contrast, the compounds depicted in Fig. 6B are those that were too short or too long and not flexible enough to accommodate as the more powerful compounds did at the site of Fig. 6A. None of them were active. These molecules can either fit into just one of the branches of the “U”, as observed with compounds **5, 8a** or **9a**, or fall into a different pocket where there is no interaction with residues from TMH 6 and 12, as observed with compounds **2**, **2b**, **2c**, **3a**–**c, 8** or **9**. The relevance of such structural determinants may be clearly illustrated by comparing the inactive compound **3c** with one of the strongest inhibitors, compound **5c**, both with very similar chemical structures, including the *ortho*-dimethoxy substitution in the quinolinone ring. The only difference between both entities is that the flexible linker to the quinolinone, the group —NHCH_2_—, is replaced by a longer and less flexible carbonyl-vinyl (—C(O)—CH═CH—) (Fig. 1). In addition to the two main groups of compounds shown in Fig. 6A,B, compound **3** with moderate activity (MEC = 10 µM, Table 1) was found to fit partially into the pattern of Fig. 6A, also touching TMH 11, a contact that is not shared by any of the other ligands (Supplementary Fig. S3, online).

While the docking revealed some clear trends in the type of interaction and sites of relevance, MD simulations provided a more detailed understanding of the nature, strength, and persistence of interactions with the key residues. The inactive compounds **5** and **8**, with the patterns shown in Fig. 6B, were selected for the studies, as well as the active compounds **3**, **5a**, **5c** and **7b**, the respective activity of which increased from low to mild to the highest (Table 1). The free energies of binding (Δ*G*^0^_b_) of these representative compounds, estimated by MD, were compared to those of the substrates and of the two reference inhibitors, verapamil, used as a positive control, and tariquidar, one of the most potent in vitro inhibitors known^2^, used as a positive control for the in silico studies. Although one substrate and tariquidar and verapamil were previously simulated^8,35^, for comparison purposes, these simulations were repeated from scratch with the novel PDB structure and using exactly the same simulation conditions as for the subject ligands. As shown in Table 2, compounds **5** and **8** were found to be unable to compete with either Rho123 or Dox, added to the fact that compound **8** went to another site of lower affinity. The Δ*G*^0^_b_ values of compounds **3**, **5a**, **5c** and **7b** (Table 2) reproduced well their inhibitory properties (Table 1). The most promising structures, **5c** and **7b**, showed similar energies to that of verapamil although not as favorable as tariquidar (Table 2). The latter comparison was not surprising, since tariquidar is known to have a low nanomolar activity^36^, despite that its high toxicity^37^, in contrast to our subject compounds, and other issues^38^ prevent it being clinically suitable.

**Table 2:** Free energies of binding from the MMPBSA analyses of the MD simulations.

Compounds	Δ*G*^0^_*b*_ (kcal/mol)
**Substrates**	
Rhodamine 123	− 23.2
Doxorubicin	− 24.2
**Subject compounds**	
**5**	− 16.7
**8**	− 22.8
**3**	− 27.1
**5a**	− 29.4
**5c**	− 33.1
**7b**	− 35.8
**Reference inhibitors**	
Verapamil	− 35.7
Tariquidar	− 38.3

**Figure 7:**
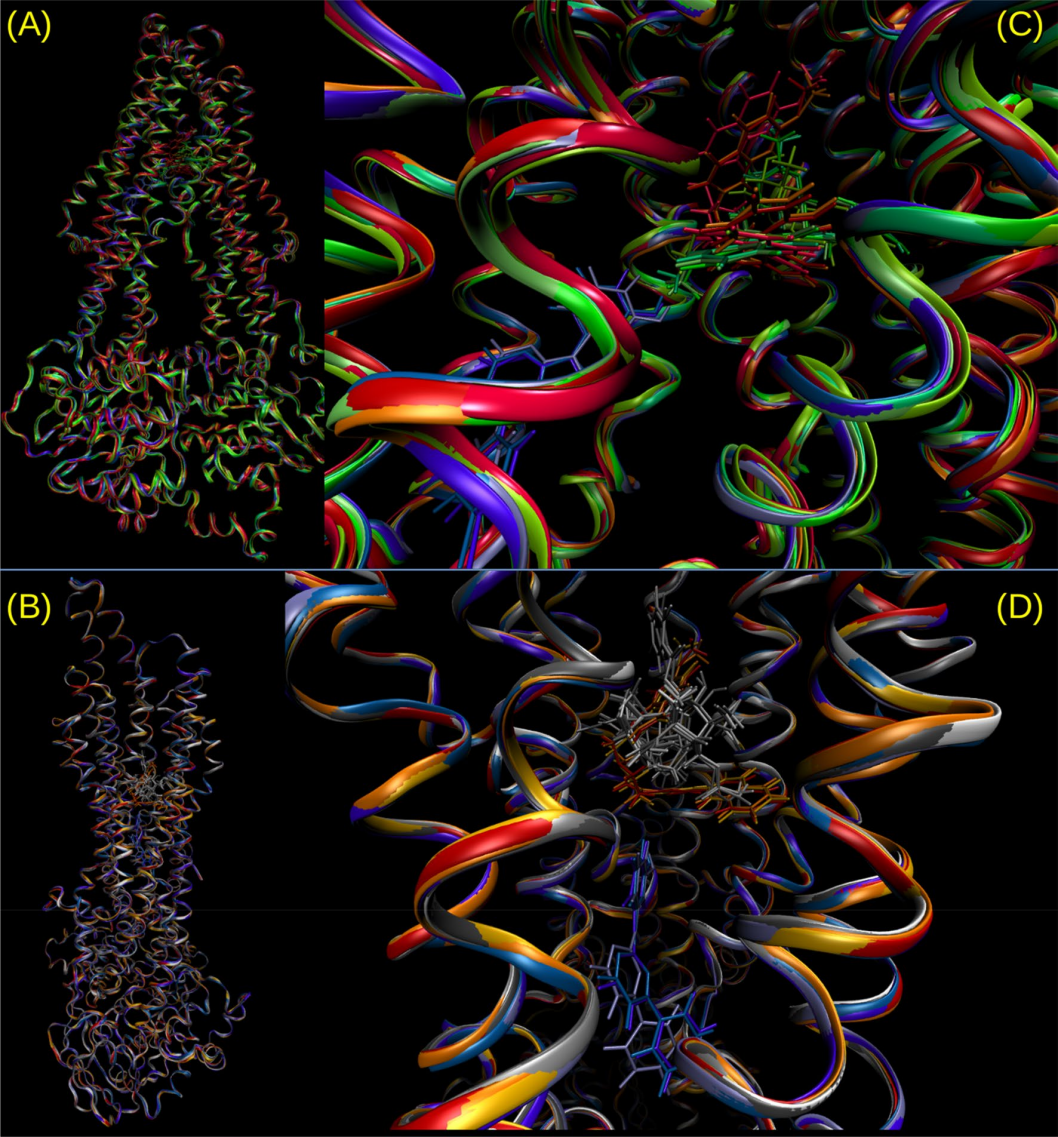
(**A**,**B**) Two views of the P-gp rotated about 80 degrees around the vertical axis; they are zoomed in (**C**,**D**. (**C**) Superimposition of the most populated clusters from a representative trajectory for compound **7b** (red tones), doxorubicin (green tones) and compound **8** (blue tones). (**D**) The same for compounds **7b** and **8** and for Taxol (gray tones).

**Figure 8:**
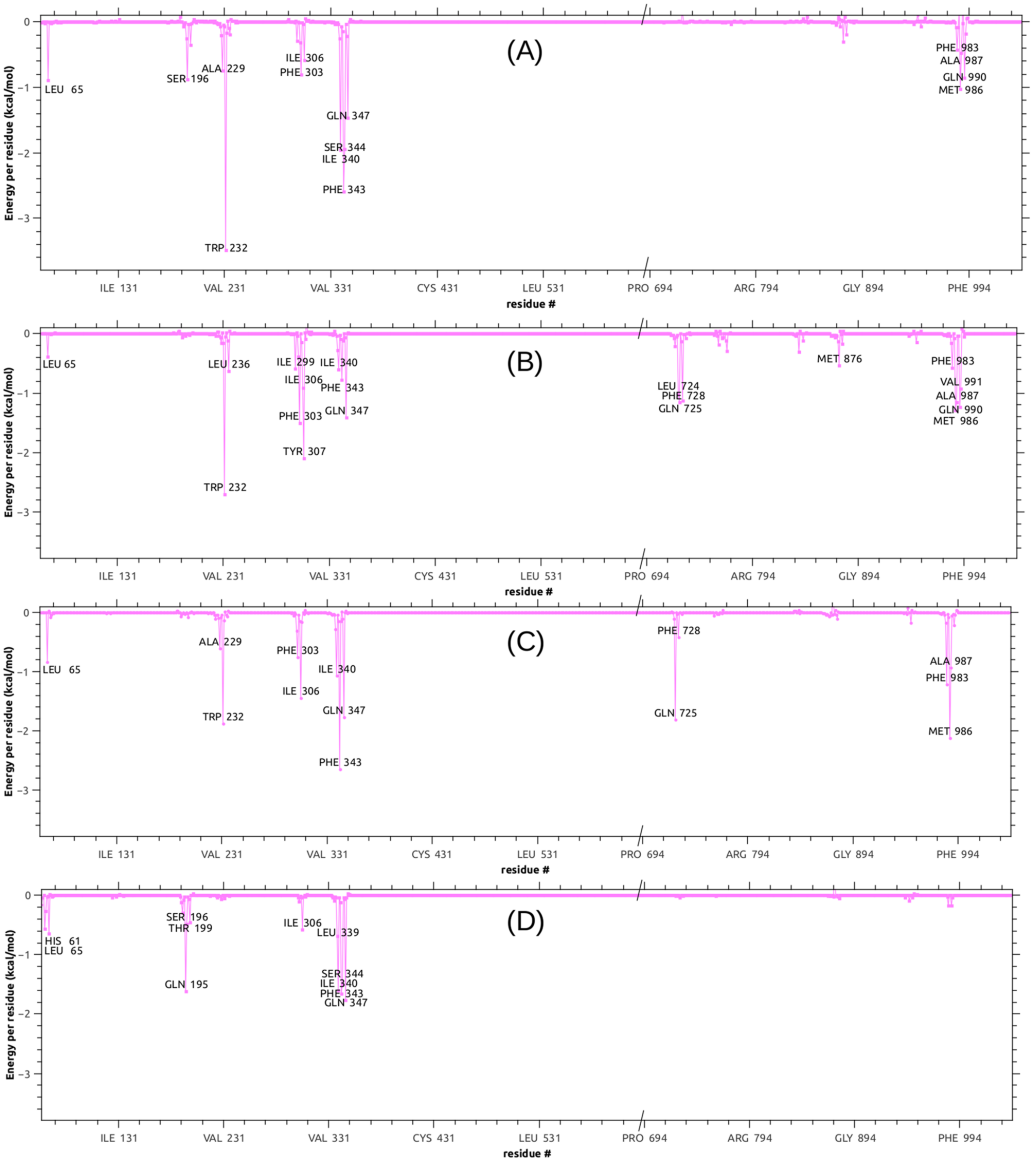
Decomposition of the MD free energy of binding in terms of per residue contribution. Residues showing the most negative peaks correspond to stronger stabilizations: (**A**) doxorubicin; (**B**) tariquidar; (**C**) compound **5c**, (**D**) compound **5**.

An analysis of the dynamic interaction of the potent compound **7b** throughout the simulation revealed that it persistently shared the region and the contacts of the known therapeutic P-gp substrates, Dox and Taxol. In contrast, the inactive compound **8**, besides its reduced affinity in terms of Δ*G*^0^_b_, stayed in a different region and had greater mobility as shown in Fig. 7, where four representative snapshots of each MD trajectory (i.e. the conformations most visited at the simulation temperature) are superimposed for Dox, Taxol and compounds **7b** and **8**. A similar behavior was observed for compounds **5a** and **5c**. Figure 8A–C shows the Δ*G*^0^_b_ components decomposition in terms of per residue energy for compound **5c**, compared to the substrate Dox and tariquidar. Most of the contacts of **5c** were shared with the top reference inhibitor. More important is the fact that most of these contacts, such as Ala229 and Trp232 from TMH 4, Phe303 and Ile306 from TMH 5, Ile340, Phe343 and Gln347 from TMH 6, and Phe983 and Met986 from TMH 12, among others, were proposed to play a key role in the binding to P-gp on both computational and experimental bases^5,8,35,39^. Compound **5c** (Fig. 8C), as well as compound **7b** (Supplementary Fig. S4, online) showed important peaks (i.e. persistent contacts) at residues from both homologous halves (TMHs 1–6 and 7–12), in particular from TMH 6 and 12, both connecting the transmembrane domain (TMD) to the nucleotide binding domains. As depicted in Fig. 8D, and as previously observed in the docking poses, the inactive compound **5** shared most of the contacts present in TMH 5 and 6 with compounds **5c** (Fig. 8C) and **7b** (Supplementary Fig. S4, online) but did not interact with those from TMH 12 or with any other from the second homologous half. This energy analysis also revealed a certain importance of contact with TMH 7 (mainly with Gln725), which is observed with compounds **5a**, **5c** and **7b** as well as with tariquidar, but not with Dox or the less active compound **3** or with the inactive compound **5 (**Fig. 8, Supplementary S2, online). Indeed, compounds **3** and **5** contact with neither TMH 7 nor TMH 12; the latter only contacted with the second half with a mild interaction with Gln946 from TMH 11 (the decomposition profile for compounds **3**, **5a**, **8** and verapamil are shown in Supplementary Fig. S4, online). Additional valuable evidence, which reinforces the relevance of these contacts, was recently published by Nosol et al.^40^. The structure of the human P-gp reported co-crystallized with vincristine (PDB entry 7A69)^40^, another chemotherapeutic substrate, showed that the hit compounds and the pose of DOX arranged in the cavity which holds the vincristine molecule. The highly persistent peaks (those labeled in Fig. 8A) for DOX are also contacts in the experimental structure of vincristine, including Leu65, Phe303, Ile306, Leu339, Ile340, Phe343, Ser344, Gln347, Phe983, Met986, Ala987 and Gln990 (see Supplementary Fig. S6, online for comparison). The same authors also reported a co-crystallized structure for tariquidar (PDB entry 7A6E), but the comparison is not plausible in this case, since tariquidar captured a two molecule-binding mode whilst our studies focused on finding inhibitors able to act at minimal concentrations. Consequently, all single molecule poses were considered^41^.

The MD simulations also revealed that the number of hydrogen bond acceptors in the molecule would not be as important as the other features discussed so far. Indeed, even though tariquidar is the species with the most H-bond acceptors, it just had a total average of about one H-bond during the simulation time; the same was observed for compounds **5c** and **7b**. On the other hand, the compound which kept the highest number of persistent H-bonds during the simulation was **3**, one of the poorest of the set (see Supplementary Fig. S5, online for a comparison of the H-bond analyses over the last 10 ns of simulations for **3**, **5c**, **7b** and tariquidar). The main interactions with the residues bearing the major peaks in Fig. 8 are hydrophobic in nature and are analyzed in more detail with QM/MM calculations.

### Charge density analysis of complexes

QTAIM calculations are very useful tools for evaluating accurately and in detail the molecular interactions which stabilize ligand-receptor complexes^42^ and can be used successfully in different biological systems^43–47^. In order to analyze the molecular interactions stabilizing the complexes of the target molecules and P-gp more quantitatively and with more detail, QM/MM calculations were performed, choosing the most representative complexes of the series. Therefore, the complexes of Dox, tariquidar and compounds **3**, **5a**, **5c**, **7b** and **8** with P-gp were further studied by charge density analysis in the context of the quantum theory of atoms in molecules (QTAIM) framework. Reduced models of complexes were constructed, containing receptor residues within the range of noncovalent interactions from ligand atoms, as reported in Material and Methods. The QTAIM topological analysis was performed by mapping the gradient vector field onto the pre-computed charge density (i.e., Δ⍴(r)) of reduced models, thus giving rise to the topological elements of the charge density, i.e., the bond critical points (BCPs) and the bond paths (BPs) that connect the interacting atoms.

**Figure 9:**
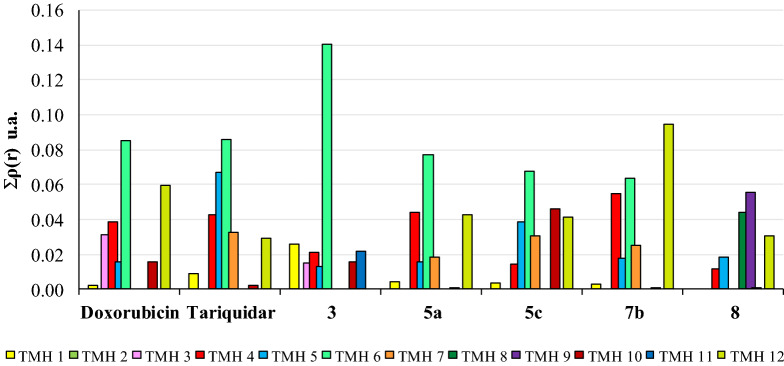
Sum of charge density values (atomic units) at the critical intermolecular binding points between P-gp and ligands with different levels of activity **3**, **5a**, **5c**, **7b** and **8**. Total interactions obtained for the different complexes are shown as a function of the different transmembrane (TMH) domains of the receptor.

Figure 9 shows the charge density values obtained for the complexes in function of the different TMH domains of P-gp, and the numerical data is shown in Supplementary Table S1, online. It is interesting to note that the complexes with Dox and the inhibitors tariquidar, **5a**, **5c** and **7b** presented a very similar binding pattern, with all the significant interactions located in residues from TMH 4–6 and 12, the most important of which were with TMH 6 and 12. It is important to highlight that tariquidar as well as compounds **5c** and **7b**, and to a lesser extent compound **5a**, showed significant interactions with TMH 7. Weaker interactions were also seen with residues located at TMH 1 and TMH 10, while no interactions with TMH 2 were observed in any complex. One exception to this motif was the case of compound **5c**, which showed somewhat significant interactions with amino acids from TMH 10. Regarding the mildly effective compound **3**, the pattern of interactions was different from that observed for the active compounds since it showed the strongest interactions with TMH 6 but lacked interactions with TMH 12. In addition, it was the only compound displaying interactions with TMH 11. The inactive compound **8** did not establish interactions with the residues of either TMH 6 or TMH 7, although it interacts with TMH 12, but with different residues to those which compounds **5c** and **7b** contacted**.** Likewise, **3** was the only compound that established interactions with TMH 8 and TMH 9. Therefore, the low or null activity of compounds **3** and **8** may be explained by their binding in widely different sites.

**Figure 10:**
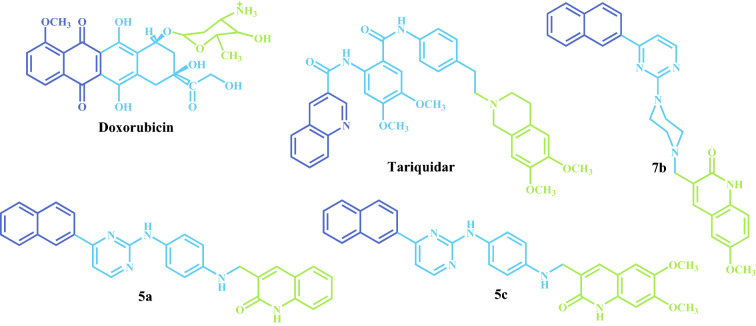
Structures of doxorubicin, tariquidar and compounds **5c**, **7b** and **5a** partitioned into three portions: moiety 1 (blue), linker (green) and moiety 2 (light blue).

**Figure 11:**
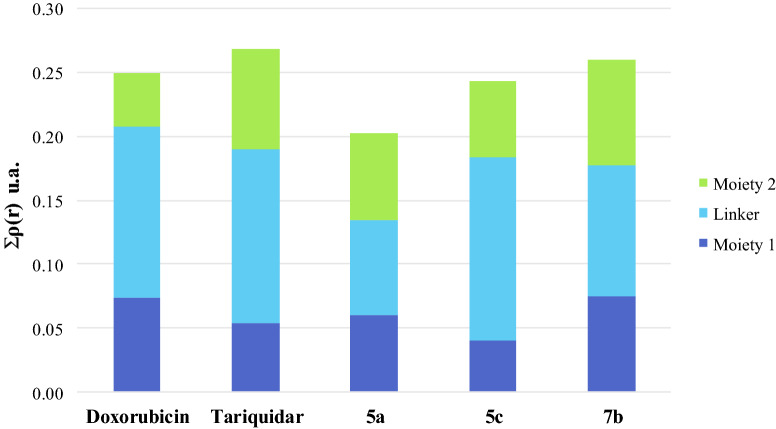
Sum of the charge density values (atomic units) at the critical intermolecular bond points between P-gp and doxorubicin, tariquidar, **5a**, **5c** and **7b**. The total of interactions established is shown according to the contributions of the three portions of each molecule.

Among the benefits of QTAIM, it has been shown that there is also a direct relationship between the charge density value in the BCP interaction and the complex interaction energy^48^. Therefore, one might expect this relationship to still hold for biomolecular complexes, i.e., the sum of charge density values from all the intermolecular BCPs should be related to the complex stability. Such a relationship concerning a particular system is important since it enables the contributions of individual functional groups to the overall anchoring strength of the ligand within the binding pocket to be measured. In order to clarify the interactions from the point of view of the target ligands, the molecules were arbitrarily divided into three portions (moiety 1, linker and moiety 2), as shown in Fig. 10. Figure 11 shows the total of interactions established according to the three portions of each molecule. Compounds **3** and **8** were not included because these interacted in a different site and, therefore, comparing their interactions with active compounds would be futile. All the complexes showed a similar pattern of interactions indicating that the three portions of the ligands established significant interactions, which stabilized the complexes. In other words, it appears that the presence of the three portions of the ligands is necessary for the activity. As expected, the interactions observed for the complex with tariquidar were the strongest, while the interactions obtained for the complex with **5a** were the weakest, and the interactions obtained for Dox, **5c** and **7b** complexes gave intermediate values. These results fully match both the experimental data and the classical MD estimations.

### Toxicity assessment of most active compounds

As the most potent molecules, the cytotoxicity of compounds **5c** and **7b** was determined against normal peripheral blood mononuclear cells (PBMC), in an effort to evaluate their safety profile. The results showed that compounds **5c** and **7b** were devoid of cytotoxicity, with 21% and 12% of inhibition on proliferation at 20 µM, respectively. In addition, compounds **5c** and **7b** did not alter the erythrocyte membrane at concentrations lower than 10 and 20 µM, respectively. The results show that these compounds may be proposed as safe candidates for developing novel agents to circumvent MDR by P-gp inhibition.

## Conclusions

In this study, 2-substituted amino-4-arylpyrimidines and their quinoline hybrids were tested for their inhibitory properties on P-gp function. Among the 31 compounds tested, 18 proved to be effective, with compounds **5c** and **7b** being the most potent to enhance Dox and Rho123 intracellular accumulation by blocking their extruding from cells. Subsequently, these compounds possess outstanding reversal activity of the P-gp-mediated Dox resistance with nanomolar potency. No influence on proliferation of PBMC nor alterations in erythrocyte membranes were observed with the most effective compounds **5c** and **7b**, thus suggesting these molecules as promising starting points for chemosensitizer agents.

Results obtained from the preliminary SAR study mainly suggest that both the presence of methoxy groups and their number were favorable for P-gp activity. Important restrictions on the length and flexibility of the labeled linker region were found to play a critical role. At least one aliphatic sp^3^ carbon of a methylene group seems to be required to enable folding between the moieties labeled 1 and 2. This feature was discussed as an example by comparing the almost identical compounds **3c** and **5c**, inactive and highly effective, respectively. The number of H-bond acceptor groups per se played no role unless they positively contributed to anchoring the inhibitor in certain poses located in specific sites and interacting with important residues.

Another interesting contribution of this work is the insight into details of certain structural aspects, which are essential for understanding the formation of the complex ligand/P-gp. Combined MM/QM/QTAIM calculations enabled us to describe the molecular interactions that stabilize the different complexes of the ligands with the protein and to determine which portion of the compounds should be changed in order to increase their affinity with P-gp. The molecular modeling study indicated that the compounds reported here bound in the same region of the active site of P-gp as that of tariquidar and Dox; however, our simulations indicated that these molecules were arranged spatially in a slightly different way. Therefore, these inhibitors are bound to some of the same amino acids as tariquidar but also to different ones.

## Materials and methods

### Chemistry

Most chemicals and solvents were used pure or dried when necessary. All starting materials were weighed and handled in air at room temperature. Thin layer chromatography (TLC) monitoring was performed on 0.2 mm pre-coated aluminum plates of silica gel (Merck 60 F_254_) and spots were visualized by UV irradiation (254 nm) to determine the progress of the reaction. If necessary, the compounds were separated by means of column chromatography on flash silica gel 60 (40–63 µm, Merck, Darmstadt, Germany). HPLC purification was further performed to ensure the > 95% purity of each compound, as determined by HPLC analysis (see details in Supplementary Information, online). High-resolution mass spectra (HRMS) analysis was performed for identity check. The characterization of synthesized compounds was performed measuring the melting points (Brastead Electrothermal 9100 melting point apparatus) and all were fitted within ± 2 °C of difference with respect to those reported^17^. ^1^H spectra were recorded in a Bruker Avance 400 spectrometer at 400 MHz at 298 K, using CDCl_3_ and DMSO-*d*_6_ as solvents, except for compounds **2a–c** and **3a–c**, which were recorded in DMSO-*d*_6_ at 393 K, and tetramethylsilane (0 ppm) as the internal reference, or the residual hydrogen of such solvents.

### General procedure for the synthesis

#### General procedure for the synthesis of 1-(4-((4-arylpyrimidin-2-yl)amino)phenyl)ethan-1-ones (2–3), *N*^1^-(4-arylpyrimidin-2-yl)benzene-1,4-diamines (4–5), 2-(4-(4-arylpyrimidin-2-yl)piperazin-1-yl)ethan-1-amines (6–7), 3/4-((4-(4-aryl)pyrimidin-2-yl)amino)phenols (8–8a)/(9–9a), 2-(4-(4-(4-aryl)pyrimidin-2-yl)piperazin-1-yl)ethan-1-amines (10-10a)

The compounds were prepared by microwave irradiation in 1,4-dioxane (1 mL) in reaction with the corresponding amino-derivative for 15 to 60 min at 120 °C, and 250 W of max. power. For the preparation of 3-((4-(4-chlorophenyl)pyrimidin-2-yl)-1-yl)oxymethyl)quinolin-2(1*H*)-ones (**11**), the 2-chloro-4-(4-chlorophenyl)pyrimidine **1a** was reacted with the 3-hydroxymethyl-6,7-dimethoxyquinolin-2-one (**IIc**, see Supplementary Fig. S2, online) under the above conditions but the addition of potassium carbonate was needed^17^.

#### General procedure for the synthesis of (*E*)-3-(3-oxo-3-(4-arylpyrimidin-2-yl)amino)phenyl)prop-1-en-1-yl)quinolin-2(1*H*)-ones (2a–c, 3a–c)

Compounds were prepared from the quinoline-3-carbaldehydes **Ia–c** and derivatives **2–3** with an excess of aqueous KOH by heating to reflux in MeOH for 3 days (see Supplementary Fig. S2, online).

#### General procedure for the synthesis of 3-(((4-((4-arylpyrimidin-2-yl)amino)phenyl)amino)methyl)quinolin-2(1*H*)-ones (4a–c, 5a–c)

The compounds were prepared from the quinoline-3-carbaldehydes **Ia**–**c**, and derivatives **4**–**5** were treated with the catalytic amount of AcOH in MeOH under reflux for 2–12 h, and then with excess of sodium borohydride at 0 °C to ambient temperature for 24 h (see Supplementary Fig. S2, online).

#### General procedure for the synthesis of 3-((4-arylpyrimidin-2-yl)piperazin-1-yl)methyl)quinolin-2(1*H*)-ones (6a–c, 7a–c)

The compounds were prepared by reaction of pyrimidines **6–7** with 3-(chloromethyl)quinolinones **IIIa–c**, in DMF using Et_3_N as promoter and heating to reflux for 8 h (see Supplementary Fig. S2, online).

### Material for biological studies

3-(4,5-Dimethyl-2-thiazolyl)-2,5-diphenyl-2*H*-tetrazolium bromide (MTT), Rho123 and Histopaque-1077 were purchased from Sigma Aldrich, (Sigma-Aldrich Co., St Louis, MO). Doxorubicin hydrochloride (99.8%, Synbias Pharma Ltd.) was obtained from Nanox Release Technology (Buenos Aires, Argentina) and a fresh solution was prepared by dissolving it in bi-distilled water. Verapamil hydrochloride 98.0%, provided by Parafarm (Buenos Aires, Argentina), was dissolved in ethanol. Measurement of the fluorescence was performed with a Life Technologies Attune-NxT flow cytometer with 96-well autosampler (Thermo Fisher Scientific, USA).

### Cell lines and cell culture

The sensitive chronic myelogenous leukemia cell line K562 and its resistant derivative, Lucena 1, were cultured in supplemented RPMI-1640 medium (Invitrogen Life Technologies, Carlsbad, CA, USA) with 10% fetal bovine serum, 2 mM L-glutamine, 100 U/mL penicillin and 100 μg/mL streptomycin (Invitrogen Life Technologies) at 37 °C in a 5% CO_2_ humidified atmosphere. Lucena 1 selectively overexpressed P-gp^49^ and its MDR was maintained by once weekly exposure to the anticancer drug, Dox at 60 nM till 4 days before the experiments^39^. Dox displayed a weak toxic effect against Lucena 1 cells, which showed 35.8-fold resistance to this drug compared to its parental K562 (IC_50_ to Dox = 22.97 ± 5.41 and 0.64 ± 0.13 µM, respectively). The Dox and Rho123 fluorescence intensity was 2.31 and 5.87-fold lower, respectively, in Lucena 1 with respect to K562. Cells used in the experiments were in the logarithmic growth phase, with a viability above 90% determined by trypan blue exclusion.

### Doxorubicin and rhodamine 123 intracellular accumulation assays

The intracellular accumulation of the fluorescent probes, Dox and Rho123, was determined by monitoring its fluorescence intensity using flow cytometry (96-well plate format), as previously reported^8^. The inhibition on the outward transport of these P-gp substrates results in an increased medium fluorescence intensity (MFI). Both cell lines, Lucena 1 and K562 at a density of 5 × 10^4^ cells/well, were seeded in 96-well culture plates containing RPMI-1640 medium and exposed to the target compounds dissolved in DMSO at the non-toxic concentration of 20 µM. Those compounds showing inhibition on P-gp at the maximum tested concentration were further evaluated at serial dilutions to determinate their MECs. Negative controls were performed with DMSO 0.5% v/v (at this concentration no differences compared to the untreated control groups containing only supplemented culture medium, were observed), while verapamil, a known P-gp inhibitor, was used as positive control. The plates were then incubated in a 5% CO_2_ incubator for 1 h at 37 °C. Afterwards, 5 μM Dox was added to each well. The other fluorescent specific substrate of P-gp, Rho123, however, was added at 500 ng/mL to additionally measure the ability of the most promising compounds to block its efflux. Subsequently, cells from both assays were washed twice with cold PBS, and 15,000 events from each sample were analyzed to determine the cell-associated fluorescence. Dead cells were eliminated by means of forward- and side-scatter parameters. The MFI of retained intracellular Dox and Rho123 were estimated using a 488 nm laser and 585/42 nm or 530/30 nm bandpass filters, respectively, and was analyzed through Flowjo software (Tree Star, Inc. Ashland, OR). The blocking of the P-gp transport function was evaluated by the parameter FIR, calculated as the ratio of the MFI of Dox or Rho123 with the addition of the likely inhibitor to the MFI of Dox or Rho123 alone.

### MTT assay

The viability of Lucena1 and K562 cells treated with the active compounds **3**, **4a–c**, **5a–c**, **6**, **6a–c**, **7**, **7a–c**, **10**, **10a** and **11** was determined by the MTT colorimetric assay as previously described^3,5^. Both cell lines, at a final density of 5 × 10^4^ cells/well, were incubated with 0.16 to 20 μM of each tested compound previously dissolved in DMSO (0.5% v/v as final concentration since at this no cytotoxicity was observed) in a 5% CO_2_ humidified atmosphere at 37 °C for 48 h. Then 0.5 mg/mL MTT in PBS was added. After additional incubation for 4 h, the formazan crystals obtained were dissolved in DMSO and the absorbance at 595 nm was read on an iMark micro-plate reader (Bio-Rad, USA). IC_50_ values for cytotoxicity were then calculated as reported^50^ using nonlinear regression by GraphPad Prism 7 (Graphpad Software, Inc., San Diego, CA, USA, www.graphpad.com).

### Doxorubicin resistance reversal assay

Based on the results obtained in the accumulation assays, the effects of the most effective compounds **5c** and **7b** on reversing Dox resistance in Lucena 1 cells were evaluated as previously described^5,8,39^. Briefly, 5 × 10^4^ Lucena 1 and K562 cells were seeded in 96-well plates containing RPMI-1640 medium with the anticancer drug Dox (3.4–431 or 0.3–34.5 µM, respectively) in the absence or presence of 0.02 to 1.25 µM of compounds **5c** and **7b**. Negative controls contained 0.5% v/v DMSO while verapamil was used as standard inhibitor. The cells were incubated at 37 °C at 5% CO_2_ for 48 h and the same protocol described above was followed. IC_50_ values of Dox were then obtained. The FR values were calculated as the ratio of the IC_50_ obtained with Dox/IC_50_ value for Dox in combination with the different concentrations of each tested compound^8,39^.

### Cytotoxicity on peripheral blood mononuclear cells and hemolysis assay

The toxic effect of the most effective compounds **5c** and **7b** on PBMC was determined by MTT assay^51^. PBMC were isolated from heparinized blood of healthy volunteers by density centrifugation using Ficoll-Hypaque separation. Ethical approval was provided by the Catholic University of Córdoba Research Ethics Board and informed consents were obtained from all donors. Briefly, 1 × 10^5^ PBMC/well were incubated with the tested compounds at concentrations ranging from 2.5 to 20 µM in the presence of 10 μg/mL PHA, or with 1% DMSO as control. After 48 h incubation, MTT was added and the same protocol described above was followed to calculate the IC_50_ values. An erythrocyte hemolysis assay was carried out following previous procedures^8^ with the compounds assayed at 2.5 to 20 µM.

### Statistical analyses

The results are expressed as mean ± SE. The statistical analysis was performed using both paired and unpaired one-tailed Student’s t test (GraphPad Prism 7.0 San Diego, CA, USA, www.graphpad.com). *P*-values ≤ 0.05 were considered as statistically significant. All experiments were repeated independently at least three times.

### Molecular modelling

#### Docking and molecular dynamics

The structures of the reference substrate (Dox), the substrate co-crystallized in the experimental P-gp structure (Taxol), the reference inhibitors tariquidar and verapamil, and the subject compounds listed in Fig. 1, were obtained by performing a conformational search (when relevant) and a full geometry optimization at the semiempirical PM6 level of theory. The structures were characterized as minima by diagonalizing the Hessian matrix and ensuring the absence of negative eigenvalues using the Gaussian 16 (Rev. A03) package^52^. Two different docking protocols were used: (1) the Autodock 4.2.6 package^53^ was used by precomputing a grid inside the whole TMD, as illustrated in Fig. 5A. The procedure has already been described and applied to a different family of compounds^5,39^. Briefly, 4000 runs of Lamarckian genetic algorithm were performed for each ligand; the relatively large number of runs was in order to take into account the large size of the docking region. The population was set at 150 individuals, up to 105 generations with 1 survivor per generation and a limit of 6 × 10^6^ energy evaluations and the remaining algorithm control parameters set to program defaults. The cluster analysis was made with 2.5 Å of RMSD^53^. (2) Similarly, within the same box, the second docking protocol was based on Autodok Vina 1.1.2–5^54^, collecting the first 10 lowest poses or those within 3 kcal/mol above the lowest, setting the ‘exhaustiveness’ parameter to 64 (default = 8) and repeating the simulations at least three times.

The recent structure of the human P-gp co-crystallized with Taxol (PDB entry 6QEX)^34^ was used. Since an experimental P-gp/chemotherapeutic complex (though at relatively low resolution) is available, the Taxol molecule was removed from the site and blind docking was assayed for both Autodock 4.2 and Vina, the latter giving the best approach. Both protocols yielded similar results. However, since the latter performed better when challenged to reproduce the experimental pose of Taxol in the X-ray structure, the discussion will be limited to the Vina results.

The most stable docked structures were used as starting geometries for the MD simulations. The ligand/protein complexes were prepared using the AMBER18^55^ leap and antechamber facilities for the parametrization of the inhibitors or substrates. The charges were obtained with the -bcc option, calling the internal AM1 of the sqm module of AMBER^55^. The general setup for the MD simulations was as follows: (I) 250 steepest descent minimization steps of the whole system, keeping the protein tightly restrained and embedded into a box of TIP3P water molecules with a minimum distance of 10 Å to each wall, and Cl-counter-ions to reach electro-neutrality as required. (II) 6500 conjugate gradient minimization steps of the whole system. (III) 100 ps slowly heating in the NTV ensemble with the protein positionally restrained in the backbone. (IV) 50 ns of simulation in the NTP ensemble, at 1 atm and 300 K. Procedures III–IV were repeated in two or three independent trajectories using the Andersen thermostat and barostat^56^. In the reasonably equilibrated system, the density fluctuated slightly around 1.019 g/mL. Due to the transmembrane nature of P-gp, a 50.0 kcal/Å^2^ harmonic restraint was kept for the backbone atoms. Electrostatic interactions were computed using the Particle Mesh Ewald (PME) method with a cutoff of 10 Å^57^. Bonds involving hydrogen atoms were constrained using the SHAKE algorithm, allowing for an integration time step of 0.002 ps. The integration was made using the pmemd.CUDA module of the AMBER18 program^55^ with the auxiliary force field GAFF for the ligands and ff14SB for the protein^58^. The trajectories were analyzed using standard AMBER cpptraj analysis tools. The free energy calculations were made using the mmpbsa module of AMBER 18 by applying Poisson–Boltzman (PB) and Generalized Born (GB) models^59^. The energy analyses were made for the last 8–12 ns of simulation as the average over at least two independent trajectories. The frames were sampled once each 5–10 saved frames (saving 1 frame every 10 ps, thus ensuring that the energy self-correlation is small enough). The clustering analyses for visualization of representative conformations and rendering of some figures were made using Chimera 1.14^60^. Most graphic rendering was prepared using VMD 1.9.3^61^.

### Topological analysis of electron density

To carry out QTAIM analysis, MD trajectories were first clustered based on the root mean square deviation (RMSD) of the heavy atoms. The representative structures from the most populated clusters were selected. Afterwards, a reduced 3D model was constructed for each compound studied, selecting only those residues that directly interact with the ligands. All amino acids found within a radius of 5 Å of distance from each ligand atom were included. The wave function of the reduced models generated at the M062X/6-31G(*d*) level of theory were computed with the Gaussian16^52^ package and were subjected to a Quantum Theory Atoms In Molecules (QTAIM) analysis^62^ using the Multiwfn software^63^. QTAIM calculations were performed in order to determine the ρ(r) values at the bond critical points (BCPs) established between each atom of the ligand and a particular atom from the backbone or side chain of an amino acid of the receptor. In order to obtain the total ρ(r) value of the interaction, the sum of the ρ(r) values of each BCP between the amino acid of either the complex or a particular TMH and each atom from the inhibitor, were performed.

## Supplementary Information

Supplementary Information.

## Data Availability

All data generated or analyzed during this study are included in this published article (and its Supplementary Information files).

## Abbreviations

BCP: Bond critical points
BP: Bond paths
MM: Molecular mechanics
QM: Quantum mechanics
