# Supplementary material for: Structure activity relationships and the binding mode of quinolinone-pyrimidine hybrids as reversal agents of multidrug resistance mediated by P-gp
Source: Sci Rep. 2021 Aug 19;11:16856. doi: 10.1038/s41598-021-96226-6 (PMC8376931; doi:10.1038/s41598-021-96226-6)
Supplement: Supplementary file 1 — Supplementary Information. [file 41598_2021_96226_MOESM1_ESM.docx]

**Structure activity relationships and the binding mode of quinolinone-pyrimidine hybrids as reversal agents of multidrug resistance mediated by P-gp**

**Jerónimo Laiolo^1^. Priscila A. Lanza^2^, Oscar Parravicini^3^, Cecilia Barbieri^2^, Daniel Insuasty^4,5^, Justo Cobo^5^, D. Mariano A. Vera^2,* * *,1^, Ricardo D. Enriz^3,* *,1^ & Maria C. Carpinella^1,*,1^**

^1^ Fine Chemical and Natural Products Laboratory, IRNASUS CONICET-UCC, Universidad Católica de Córdoba, Avda. Armada Argentina 3555, Córdoba X5016DHK, Argentina.^2^ Department of Chemistry, College of Exact and Natural Sciences, Universidad Nacional de Mar del Plata - QUIAMM – INBIOTEC CONICET, Funes 3350, Mar del Plata, Argentina. ^3^ Faculty of Chemistry, Biochemistry and Pharmacy, Universidad Nacional de San Luis; IMIBIO-SL, Ejército de los Andes 950, San Luis 5700, Argentina. ^4^ Department of Chemistry and Biology, Universidad del Norte, Km 5 vía Puerto Colombia, Barranquilla 081007, Colombia. ^5^ Department of Inorganic and Organic Chemistry, Universidad de Jaén, Campus Las Lagunillas s/n, 23071, Jaén, Spain.

* Corresponding author. email: [ceciliacarpinella@ucc.edu.ar](mailto:ceciliacarpinella@ucc.edu.ar) (M.C. Carpinella),

** Corresponding author. email: [danielenriz@gmail.com](mailto:danielenriz@gmail.com) (R.D. Enriz),

*** Corresponding author. email: [dmavera@yahoo.com](mailto:dmavera@yahoo.com) (D.M.A. Vera).

^1^ These authors share equal senior authorship.

**Table S1.**

Sum of charge density values (∑ρ(r) atomic units) at the bond critical points between P-gp and ligands with different levels of activity. These values are presented as a function of the different transmembrane (TMH) domains of the receptor.

| TMH | Compound | | | | | | |
| --- | --- | --- | --- | --- | --- | --- | --- |
|  | doxorubicin | tariquidar | **3** | **5a** | **5c** | **7b** | **8** |
| TMH 1 | 0.002593 | 0.008888 | 0.025958 | 0.004151 | 0.003587 | 0.002870 | - |
| TMH 2 | - | - | - | - | - | - | - |
| TMH 3 | 0.031275 | - | 0.015295 | - | - | - | - |
| TMH 4 | 0.038774 | 0.042914 | 0.021078 | 0.043760 | 0.014509 | 0.054797 | 0.011676 |
| TMH 5 | 0.015611 | 0.066937 | 0.012882 | 0.015997 | 0.038896 | 0.017987 | 0.018174 |
| TMH 6 | 0.085523 | 0.085568 | 0.140271 | 0.077235 | 0.067866 | 0.063648 | - |
| TMH 7 | - | 0.032670 | - | 0.018476 | 0.030696 | 0.025487 | - |
| TMH 8 | - | - | - | - | - | - | 0.043847 |
| TMH 9 | - | - | - | - | - | - | 0.055857 |
| TMH 10 | 0.015653 | 0.002017 | 0.015958 | 0.000133 | 0.046388 | 0.000616 | 0.000587 |
| TMH 11 | - | - | 0.022084 | - | - | - | - |
| TMH 12 | 0.059860 | 0.029192 | - | 0.042835 | 0.041567 | 0.094637 | 0.030535 |
| *TOTAL* | *0.249289* | *0.268186* | *0.253526* | *0.202587* | *0.243509* | *0.260042* | *0.160676* |

TMH: transmembrane helice.

**Figure S1.** Synthesis of quinoline precursors **I**-**III**(**a**-**c**) [^1^](#_ENREF_1).

**Figure S2.** Synthesis of 2-substituted amino-4-arylpyrimidines **2**-**11** and **8a**-**10a** and their quinoline hybrids **2**-**7**(**a**-**c**) [^2^](#_ENREF_2).


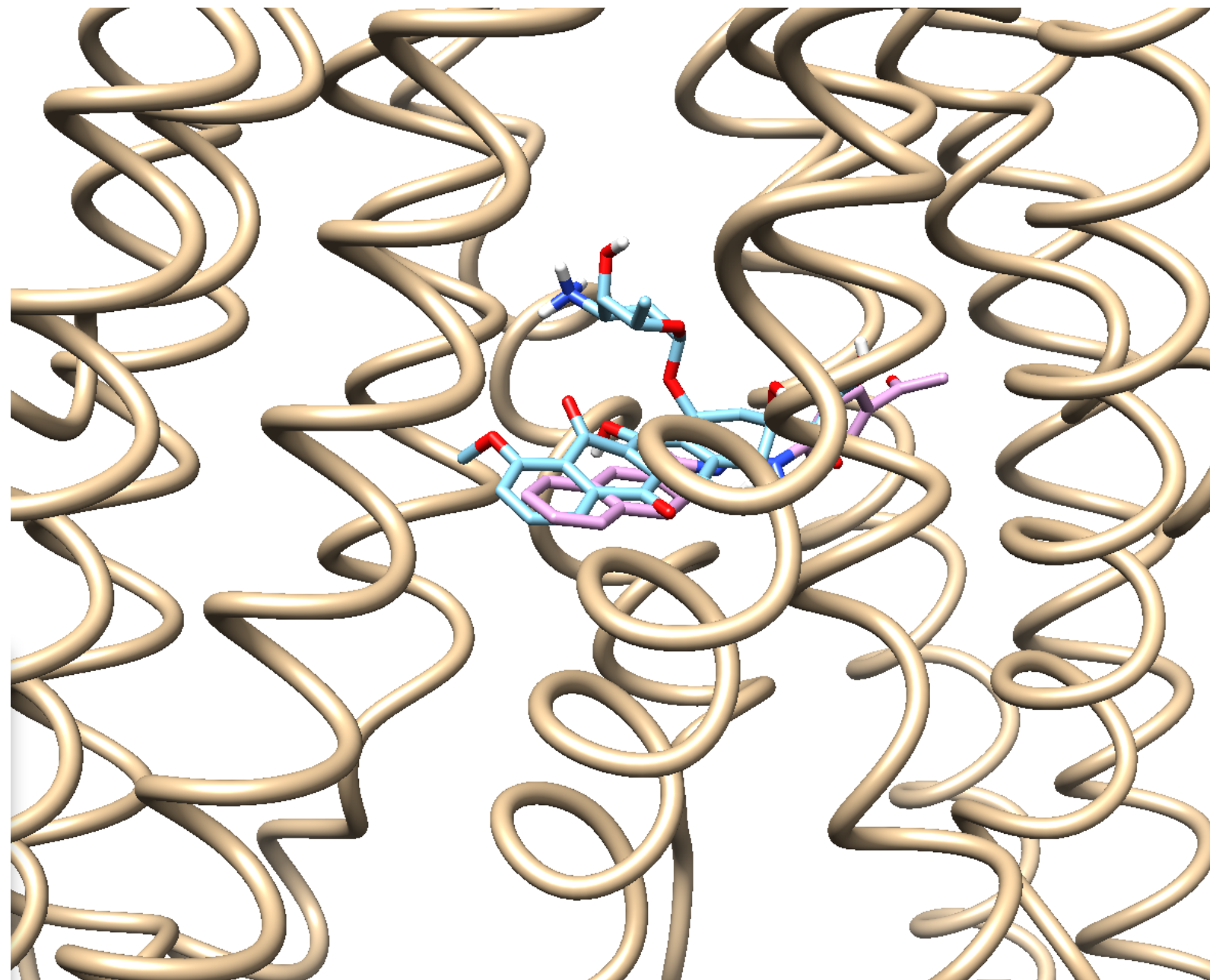


**Figure S3**. Superimposed structures of the most stable docked poses of doxorubicin (cyan) and compound **3** (pink).


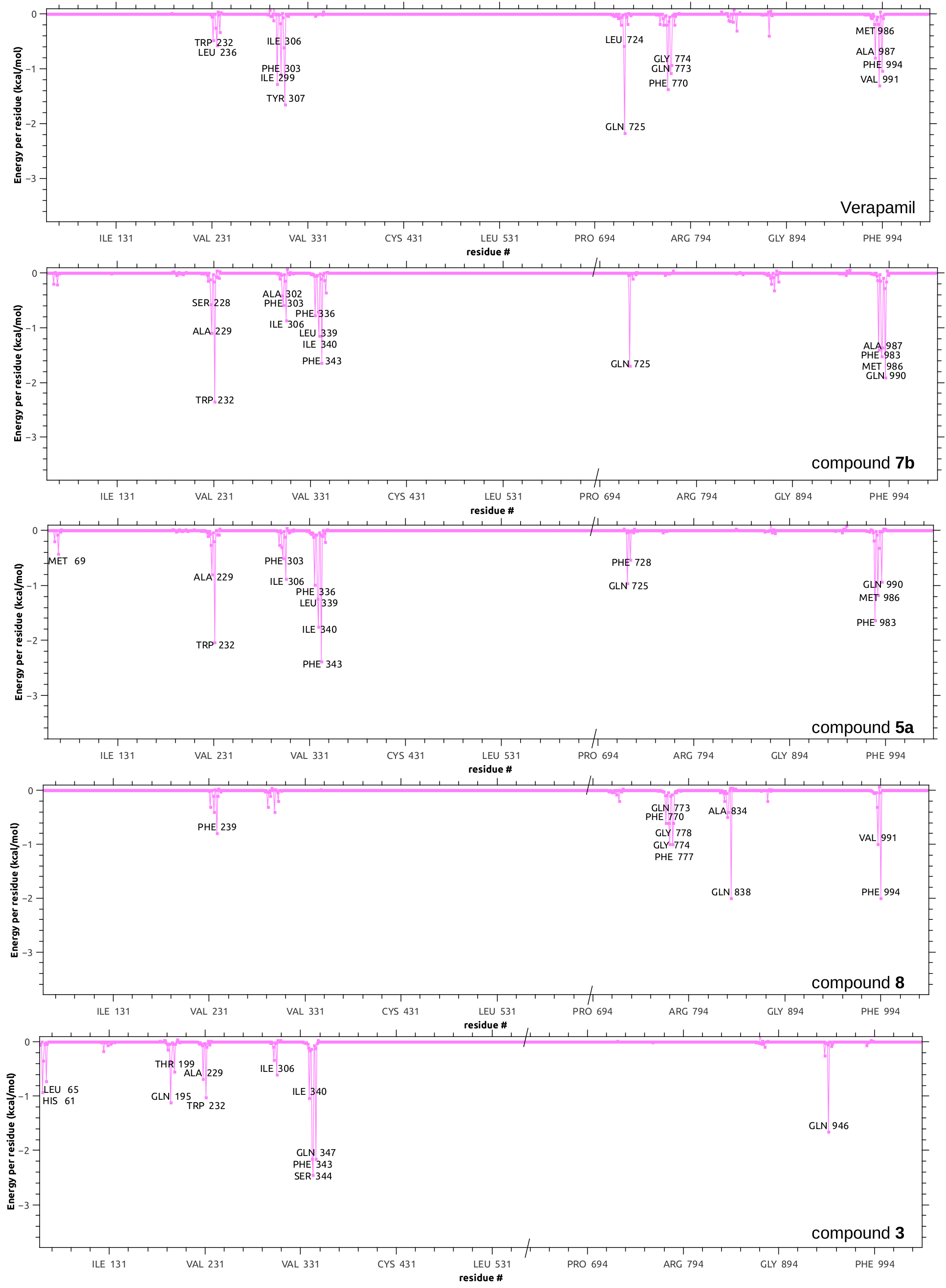
**Figure S4**. Energy decomposition on a per residue basis, same as main text in Fig. 8.


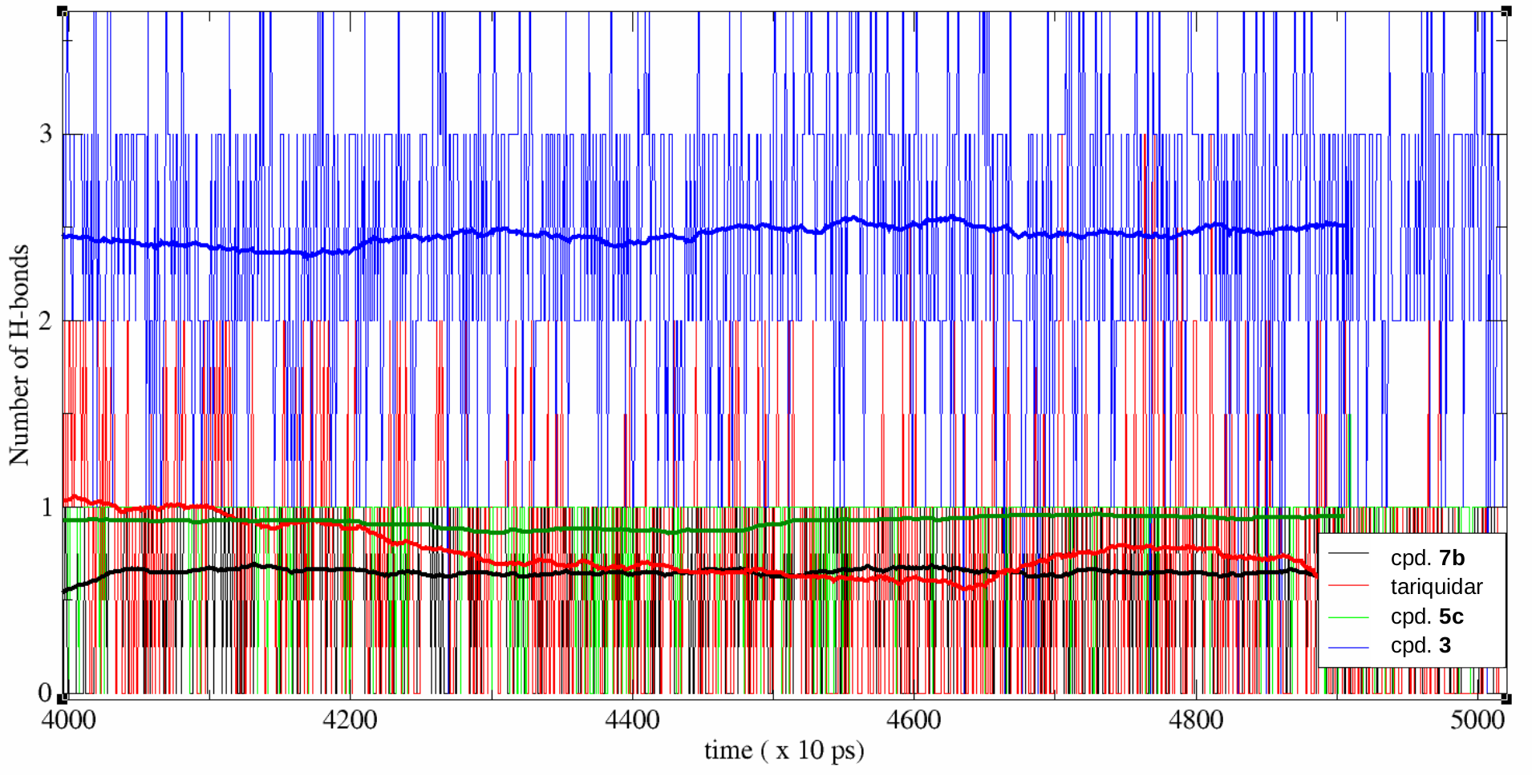


**Figure S5**. Number of H-bonds in time for tariquidar and the subject compounds **3**, **5c** and **7b**. Running average over 200 ps in bold lines.

**
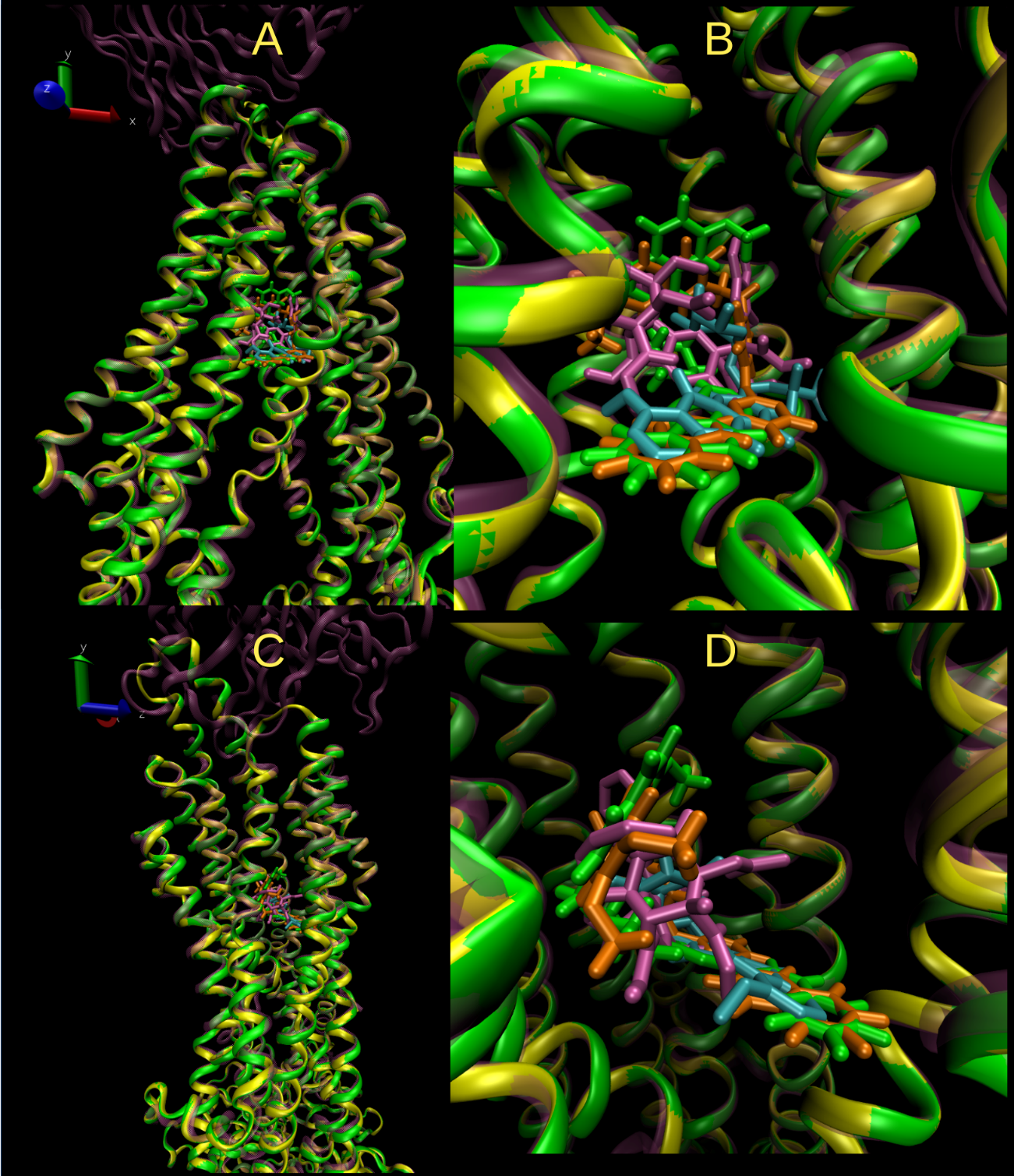
**

**Figure S6**. A-B) Superimposition of the calculated poses of doxorubicin (cyan), compound **5c** (orange), compound **7b** (green) and the experimental structure of the antitumoral drug, vincristine (violet, PDB entry 7A69). C-D) Rotated view according to the orientation of the *xyz* axis at the upper left corner of each row.

**Spectroscopic Data**.

Melting points were collected using a Brastead Electrothermal 9100 melting point apparatus, and the acquired data are uncorrected. Chemical shifts (*δ*) are given in ppm and coupling constants (J) are given in Hz. The following abbreviations are used for multiplicities: s = singlet, d = doublet, t = triplet, q= quartet, and m = multiplet. HPLC analysis was performed on an Agilent-1200 instrument equipped with a Agilent ZORBAX Extend-C18 (2.1 mm x 50 mm x 1.8 µm) PN 727700-902 column eluted with a gradient of CH_3_CN/H_2_O (10%, with 0.1% of formic acid) to CH_3_CN (100% with 0.1% of formic acid) at a flow rate of 0.4 mL/min. High-resolution mass spectra (HRMS) were recorded on an Agilent Technologies QTOF 6520B spectrometer via an electrospray ionization (ESI).

*1-(4-((4-(4-chlorophenyl)pyrimidin-2-yl)amino)phenyl)ethan-1-one* (**2**). Yellow solid, mp: 201-202 °C, ^1^H NMR (400 MHz, DMSO-*d_6_*) δ 2.49 (s, 3H, CH_3_), 7.49 (d, J = 5.3 Hz, 1H, CH), 7.60 (d, J = 8.8 Hz, 2H, H-Ar), 7.91 (d, J = 9.1 Hz, 1H, H-Ar), 7.91 (d, J = 9.1 Hz, 1H, H-Ar), 8.18 (d, J = 8.8 Hz, 2H, H-Ar), 8.61 (d, J = 5.2 Hz, 1H, CH), 10.13 (s, 1H, NH). ESI-QTOF (positive ionization) M+H calcd. for C_18_H_14_ClN_3_O, 324.0904; found, 324.0897.

**Figure S7.** HPLC chromatogram of compound **2**, C_18_H_14_ClN_3_O

*1-(4-((4-(Naphthalen-2-yl)pyrimidin-2-yl)amino)phenyl)ethenone* (**3**). Yellow solid, mp: 130-131 °C, ^1^H NMR (400 MHz, DMSO-*d_6_*) δ 2.53 (s, 3H, CH_3_), 7.62 - 7.56 (m, 2H), 7.65 (d, J = 5.2 Hz, 1H), 8.15 - 7.89 (m, 7H), 8.30 (dd, J = 8.6, 1.7 Hz, 1H), 8.66 (d, J = 5.2 Hz, 1H), 8.78 (s, 1H), 10.18 (s, 1H, NH). ESI-QTOF (positive ionization) M+H calcd. for C_22_H_16_N_3_O, 340.1450 found, 340.1442.

**Figure S8.** HPLC chromatogram of compound **3**, C_22_H_16_N_3_O

*N^1^-(4-(4-Chlorophenyl)pyrimidin-2-yl)benzene-1,4-diamine* (**4**)*.* Yellow solid, mp: 161 °C, ^1^H NMR (400 MHz, DMSO-*d_6_*) δ 4.76 (s, 2H, NH_2_), 6.55 (d, J = 8.6 Hz, 2H, H-Ar), 7.23 (d, J = 5.1 Hz, 1H, CH), 7.37 (d, J = 8.6 Hz, 2H, H-Ar), 7.58 (d, J = 8.5 Hz, 2H, H-Ar), 8.12 (d, J = 8.5 Hz, 2H, H-Ar), 8.43 (d, J = 5.1 Hz, 1H, CH), 9.14 (s, 1H, NH). ESI-QTOF (positive ionization) M+H calcd. for C_16_H_13_ClN_4_, 297.0906; found, 297.0902.

**Figure S9.** HPLC chromatogram of compound **4**, C_16_H_13_ClN_4_

*N^1^-(4-(naphthalen-2-yl)pyrimidin-2-yl)benzene-1,4-diamine* (**5**)*.* Yellow solid, mp: 130-131 °C, ^1^H NMR (400 MHz, DMSO-*d_6_*) δ 4.76 (s, 2H, NH_2_), 6.58 (d, J = 8.8 Hz, 2H, H-Ar), 7.39 (d, J = 5.2 Hz, 1H, CH), 7.44 (d, J = 8.8 Hz, 2H, H-Ar), 7.68 - 7.52 (m, 2H), 8.00 - 7.93 (m, 1H), 8.09 - 8.00 (m, 2H), 8.24 (dd, J = 8.6, 1.8 Hz, 1H, CH), 8.47 (d, J = 5.2 Hz, 1H, CH), 8.70 (d, J = 1.2 Hz, 1H, CH), 9.18 (s, 1H, NH). ESI-QTOF (positive ionization) M+H calcd. for C_20_H_16_N_4_, 313,1453; found, 313,1446.

**Figure S10.** HPLC chromatogram of compound **5**, C_20_H_16_N_4_

*4-(4-chlorophenyl)-2-(piperazin-1-yl)pyrimidine* (**6**). White solid, mp: 89-90 °C, ^1^H NMR (400 MHz, CDCl_3_) δ 3.28 - 2.74 (m, 4H), 4.17 - 3.73 (m, 4H), 6.88 (d, J = 5.1 Hz, 1H, CH), 7.42 (d, J = 8.7 Hz, 2H, H-Ar), 7.98 (d, J = 8.7 Hz, 2H, H-Ar), 8.37 (d, J = 5.1 Hz, 1H, CH), NH not detected. ESI-QTOF (positive ionization) M+H calcd. for C_14_H_15_ClN_4_, 275.1063; found, 275.1058.

**Figure S11.** HPLC chromatogram of compound **6**, C_14_H_15_ClN_4_

*4-(naphthalen-2-yl)-2-(piperazin-1-yl)pyrimidine* (**7**). White solid, mp: 112 - 113 C, ^1^H NMR (400 MHz, CDCl_3_) δ 3.05 (br, 4H, CH_2_), 4.00 (br, 4H, CH_2_), 7.11 (d, J = 5.1 Hz, 1H, CH), 7.71 - 7.45 (m, 2H), 8.06 - 7.86 (m, 3H), 8.19 (dd, J = 8.5, 1.4 Hz, 1H, CH), 8.44 (d, J = 5.0 Hz, 1H, CH), 8.56 (s, 1H, CH), NH not detected. ESI-QTOF (positive ionization) M+H calcd. for C_18_H_18_N_4_, 291.1610; found, 291.1604.

******

**Figure S12.** HPLC chromatogram of compound **7**, C_18_H_18_N_4_

*3-((4-(4-chlorophenyl)pyrimidin-2-yl)amino)phenol* (**8**). Beige solid. 83% yield, mp: 162 °C, ^1^H NMR (400 MHz, DMSO-*d_6_*) δ 7.06 (t, J = 8.1 Hz, 1H, CH), 7.21 (ddd, J = 8.1, 1.9, 0.8 Hz, 1H, CH), 7.36 (t, J = 2.2 Hz, 1H, CH), 7.38 (d, J = 5.2 Hz, 1H, CH), 7.60 (d, J = 8.7 Hz, 2H, H-Ar), 7.60 (d, J = 8.7 Hz, 2H, H-Ar), 8.54 (d, J = 5.2 Hz, 1H, CH), 9.25 (s, 1H, CH), 9.56 (s, 1H, NH). ESI-QTOF (positive ionization) M+H calcd. for C_16_H_12_ClN_3_O, 298.0747; found, 298.0743.

**Figure S13.** HPLC chromatogram of compound **8**, C_16_H_12_ClN_3_O

*3-((4-(naphthalen-2-yl)pyrimidin-2-yl)amino)phenol* (**8a**). Beige solid, mp: 158 °C; ^1^H NMR (400 MHz, DMSO-*d_6_*) δ 6.41 (dd, J = 7.9, 1.3 Hz, 1H, CH), 7.10 (t, J = 8.0 Hz, 1H, CH), 7.28 (d, J = 7.9 Hz, 1H, CH), 7.45 (s, 1H, CH), 7.53 (d, J = 5.2 Hz, 1H, CH), 7.70 - 7.55 (m, 2H), 8.02 – 7.92 (m, 1H, CH), 8.06 (d, J = 8.3 Hz, 2H), 8.29 (d, J = 8.5 Hz, 1H, CH), 8.57 (d, J = 5.2 Hz, 1H, CH), 8.76 (s, 1H, CH), 9.29 (s, 1H, NH), 9.59 (s, 1H, OH). ESI-QTOF (positive ionization) M+H calcd. for C_20_H_15_N_3_O, 314.1293; found, 314.1287.

******

**Figure S14.** HPLC chromatogram of compound **8a**, C_20_H_15_N_3_O

*4-((4-(4-chlorophenyl)pyrimidin-2-yl)amino)phenol* (**9**)*.* Beige solid, mp: 152 °C, ^1^H NMR (400 MHz, CDCl_3_) δ 6.02 (s, OH), 6.81 (d, J = 8.8 Hz, 2H, H-Ar), 7.06 (d, J = 5.3 Hz, 1H, CH), 7.15 (s, 1H, NH), 7.49 - 7.36 (m, 4H, H-Ar), 7.97 (d, J = 8.7 Hz, 2H, H-Ar), 8.41 (d, J = 5.3 Hz, 1H, CH). ESI-QTOF (positive ionization) M+H calcd. for C_16_H_12_ClN_3_O, 298.0747; found, 298.0742.

**Figure S15.** HPLC chromatogram of compound **9**, C_16_H_12_ClN_3_O

*4-((4-(naphthalen-2-yl)pyrimidin-2-yl)amino)phenol* (**9a**). Beige solid, mp: 187°C, ^1^H NMR (400 MHz, DMSO-*d_6_*) δ 6.75 (d, J = 8.2 Hz, 2H, H-Ar), 7.45 (d, J = 5.2 Hz, 1H, CH), 7.67 - 7.52 (m, 4H), 8.01 - 7.91 (m, 1H, CH), 8.05 (d, J = 8.2 Hz, 2H, H-Ar), 8.25 (dd, J = 8.7, 1.8 Hz, 1H, CH), 8.50 (d, J = 5.2 Hz, 1H, CH), 8.71 (s, 1H, CH), 9.04 (s, 1H, NH), 9.35 (s, 1H, OH). ESI-QTOF (positive ionization) M+H calcd. for C_20_H_15_N_3_O, 314.1293; found, 314.1288.

**Figure S16.** HPLC chromatogram of compound **9a**, C_20_H_15_N_3_O

*2-(4-(4-(4-chlorophenyl)pyrimidin-2-yl)piperazin-1-yl)ethan-1-amine* (**10**)_._ White solid, mp: 93 °C, ^1^H NMR (400 MHz, CDCl_3_) δ 1.68 (s, 2H, NH_2_), 2.49 (t, J = 6.1 Hz, 2H, CH_2_), 2.65 – 2.52 (m, 4H, CH_2_), 2.86 (t, J = 6.1 Hz, 2H, CH_2_), 4.09 - 3.82 (m, 4H, CH_2_), 6.88 (d, J = 5.1 Hz, 1H, CH), 7.43 (d, J = 8.6 Hz, 2H, H-Ar), 7.98 (d, J = 8.6 Hz, 2H, H-Ar), 8.37 (d, J = 5.1 Hz, 1H, CH). ESI-QTOF (positive ionization) M+H calcd. for C_16_H_20_ClN_5_, 318,1485; found, 318,1480.

**

**Figure S17.** HPLC chromatogram of compound **10**, C_16_H_20_ClN_5_

*2-(4-(4-(naphthalen-2-yl)pyrimidin-2-yl)piperazin-1-yl)ethan-1-amine* (**10a**). White solid, mp: 107 C, ^1^H NMR (400 MHz, CDCl_3_) δ 1.63 (s, 2H, NH_2_), 2.50 (t, J = 6.1 Hz, 2H, CH_2_), 2.64 - 2.53 (m, 4H, CH_2_), 2.86 (t, J = 6.1 Hz, 2H, CH_2_), 4.06 - 3.88 (m, 4H, CH_2_), 7.07 (d, J = 5.2 Hz, 1H, CH), 7.57 - 7.47 (m, 2H), 7.89 - 7.84 (m, 1H, CH), 7.92 (d, J = 8.7 Hz, 1H, CH), 7.99 - 7.93 (m, 1H, CH), 8.16 (dd, J = 8.6, 1.8 Hz, 1H, CH), 8.41 (d, J = 5.2 Hz, 1H, CH), 8.52 (d, J = 1.2 Hz, 1H, CH). ESI-QTOF (positive ionization) M+H calcd. for C_20_H_23_N_5_, 334.2032; found, 334.2025.

******

**Figure S18.** HPLC chromatogram of compound **10a**, C_20_H_23_N_5_

*3-(((4-(4-chlorophenyl)pyrimidin-2-yl)oxy)methyl)-6,7-dimethoxyquinolin-2(1H)-one* (**11**)**.** Beige solid, mp: 206 °C, ^1^H NMR (400 MHz, DMSO-*d_6_*) δ 3.80 (s, 3H, OCH_3_), 3.82 (s, 3H, OCH_3_), 4.11 (s, 2H, OCH_2_), 6.95 (s, 1H, Quin-H), 7.18 (s, 1H, Quin-H), 7.27 (d, J = 3.5 Hz, 1H, CH), 7.57 (d, J = 8.6 Hz, 2H, Ar-H), 8.07 (s, 1H, Quin-H), 8.16 (d, J = 8.5 Hz, 2H, Ar-H), 8.48 (d, J = 4.9 Hz, 1H, CH), 12.07 (s, 1H, NH). ESI-QTOF (positive ionization) M+H calcd. for C_22_H_18_ClN_3_O_4_, 424,1064; found, 424.1058.

**Figure S19.** HPLC chromatogram of compound **11**, C_22_H_18_ClN_3_O_4_

*(E)-3-(3-(4-((4-(4-chlorophenyl)pyrimidin-2-yl)amino)phenyl)-3-oxoprop-1-en-1-yl)quinolin-2(1H)-one* (**2a**). Yellow solid, mp: ˃300 °C, ^1^H NMR (400 MHz, DMSO-*d_6_*) δ 7.21 (t, J = 7.5 Hz, 1H, Quin-H), 7.38 (d, J = 8.2 Hz, 1H, Quin-H), 7.43 (d, J = 5.2 Hz, 1H, CH), 7.53 (t, J = 7.7 Hz, 1H, Quin-H), 7.60 (d, J = 8.6 Hz, 2H, Ar-H), 7.71 (d, J = 7.9 Hz, 1H, Quin-H), 7.75 (d, J = 15.7 Hz, 1H, =CH), 8.09 - 7.97 (m, 4H, Ar-H), 8.17 (d, J = 8.6 Hz, 2H, Ar-H), 8.23 (d, J = 15.7 Hz, 1H, =CH), 8.40 (s, 1H, Quin-H), 8.62 (d, J = 5.2 Hz, 1H, CH), 9.66 (s, 1H, NH), 11.56 (s, 1H, NH). ESI-QTOF (positive ionization) M+H calcd. for C_28_H_19_ClN_4_O_2_, 479.1275; found, 479.1262.

**Figure S20.** HPLC chromatogram of compound **2a**, C_28_H_19_ClN_4_O_2_

*(E)-3-(3-(4-((4-(4-chlorophenyl)pyrimidin-2-yl)amino)phenyl)-3-oxoprop-1-en-1-yl)-6-methoxyquinolin-2(1H)-one* (**2b**). Yellow solid, mp: ˃300 °C, ^1^H NMR (400 MHz, DMSO-*d_6_*) δ 3.84 (s, 3H, OCH_3_). 7.26 - 7.16 (m, 2H, Quin-H), 7.33 (d, J = 8.8 Hz, 1H, Quin-H), 7.43 (d, J = 5.2 Hz, 1H, CH), 7.59 (d, J = 8.5 Hz, 2H, Ar-H), 7.74 (d, J = 15.6 Hz, 1H, =CH), 8.09 - 7.99 (m, 4H, Ar-H), 8.17 (d, J = 8.5 Hz, 1H, Ar-H), 8.23 (d, J = 15.6 Hz, 1H, =CH), 8.35 (s, 1H, Quin-H), 8.61 (d, J = 5.2 Hz, 1H, CH), 9.65 (s, 1H, NH), 11.45 (s, 1H, NH). ESI-QTOF (positive ionization) M+H calcd. for C_29_H_21_ClN_4_O_3_, 509,1380; found, 509.1371.

**Figure S21.** HPLC chromatogram of compound **2b**, C_29_H_21_ClN_4_O_3_

*(E)-3-(3-(4-((4-(4-chlorophenyl)pyrimidin-2-yl)amino)phenyl)-3-oxoprop-1-en-1-yl)-6,7-dimethoxyquinolin-2(1H)-one* (**2c**). Yellow solid, mp: ˃300 °C, ^1^H NMR (400 MHz, DMSO-*d_6_*) δ 4.02 (s, 3H, OCH_3_), 4.05 (s, 3H, OCH_3_), 6.94 (s, 1H, Quin-H), 7.21 (s, 1H, Quin-H), 7.55 (d, J = 4.8 Hz, 1H, CH), 7.65 (d, J = 7.9 Hz, 2H, Ar-H), 7.76 (d, J = 14.6 Hz, 1H, =CH), 8.02 (d, J = 8.5 Hz, 2H, Ar-H), 8.05 (d, J = 8.5 Hz, 2H, Ar-H), 8.20 (d, J = 7.9 Hz, 2H, Ar-H), 8.27 (d, J = 14.6 Hz, 1H, =CH), 8.39 (s, 1H, Quin-H), 8.60 (d, J = 4.8 Hz, 1H, CH), 10.05 (s, 1H, NH), 11.65 (s, 1H, NH). ESI-QTOF (positive ionization) M+H calcd. for C_30_H_23_ClN_4_O_4_, 539.1481; found, 539.1477.

**Figure S22.** HPLC chromatogram of compound **2c**, C_30_H_23_ClN_4_O_4_

*(E)-3-(3-(4-((4-(naphthalen-2-yl)pyrimidin-2-yl)amino)phenyl)-3-oxoprop-1-en-1-yl)quinolin-2(1H)-one* (**3a**). Yellow solid, mp: ˃300 °C, ^1^H NMR (400 MHz, DMSO-*d_6_*) δ 7.27 (t, J = 7.2 Hz, 1H, Quin-H). 7.39 (d, J = 8.1 Hz, 1H, Quin-H), 7.59 (t, J = 7.3 Hz, 1H, Quin-H), 7.68 - 7.62 (m, 2H), 7.72 (d, J = 5.1 Hz, 1H, CH), 7.75 (d, J = 7.8 Hz, 1H, Ar-H), 7.84 (d, J = 15.5 Hz, 1H, =CH), 8.03 (d, J = 4.9 Hz, 1H Ar-H), 8..08 - 8.21 (m, 6H, Ar-H), 8.36 (d, J = 7.1 Hz, 1H, Quin-H), 8.38 (d, J = 15.1 Hz, 1H, =CH), 8.64 (s, 1H, Quin-H), 8.72 (d, J = 4.9 Hz, 1H, NH), 8.83 (s, 1H, NH). ESI-QTOF (positive ionization) M+H calcd. for C_32_H_22_N_4_O_2_, 495.1821; found, 495.1814.

**Figure S23.** HPLC chromatogram of compound **3a**, C_32_H_22_N_4_O_2_

*(E)-6-methoxy-3-(3-(4-((4-(naphthalen-2-yl)pyrimidin-2-yl)amino)phenyl)-3-oxoprop-1-en-1-yl)quinolin-2(1H)-one* (**3b**). Yellow solid, mp: ˃300 °C, ^1^H NMR (400 MHz, DMSO-*d_6_*) δ 3.84 (s, 3H, OCH_3_), 7.25 (s, 1H, Quin-H), 7.26 (d, J = 7.1Hz, 1H, Quin-H), 7.33 (d, J = 9.7 Hz, 1H, Ar-H ), 7.70 - 7.59 (m, 2H, Ar-H), 7.73 (d, J = 5.2 Hz, 1H, CH), 7.83 (d, J = 15.5 Hz, 1H, =CH), 8.08 - 8.00 (m, 1H, Ar-H), 8.21 - 8.11 (m, 6H, Ar-H), 8.36 (d, J = 7.0 Hz, 1H, Quin-H) 8.39 (d, J = 15.4 Hz, 1H, =CH), 8.58 (s, 1H, Ar-H), 8.73 (d, J = 5.2 Hz, 1H, CH), 8.84 (s, 1H, Quin-H), 10.30 (s, 1H, NH), 12.0 (s, 1H, NH). ESI-QTOF (positive ionization) M+H calcd. for C_33_H_24_N_4_O_3_, 525.1927; found, 525.1910.

**Figure S24.** HPLC chromatogram of compound **3b**, C_33_H_24_N_4_O_3_

*(E)-6,7-dimethoxy-3-(3-(4-((4-(naphthalen-2-yl)pyrimidin-2 yl)amino) phenyl)-3-oxoprop-1-en-1-yl)quinolin-2(1H)-one* (**3c**). Yellow solid, mp: ˃300 °C, ^1^H NMR (400 MHz, DMSO-*d_6_*) δ 3.84 (s, 3H, OCH_3_). 3.87 (s, 3H, OCH_3_), 6.92 (s, 1H, Quin-H), 7.20 (s, 1H, Quin-H),7.69 - 7.59 (m, 2H, Ar-H), 7.72 (d, J = 5.2 Hz, 1H, CH), 7.81 (d, J = 15.4 Hz, 1H, =CH), 8.04 (m, 2H, Ar-H), 8.18 - 8.10 (m, 6H, Ar-H, Ar-H), 8.35 (d, J = 15.5 Hz, 1H, =CH), 8.36 (d, J = 8.8 Hz, 1H, Ar-H), 8.45 (s, 1H, Quin-H), 8.72 (d, J = 5.2 Hz, 1H, CH), 8.83 (s, 1H, NH), 10.26 (s, 1H, NH). ESI-QTOF (positive ionization) M+H calcd. for C_34_H_26_N_4_O_4_, 555.2032; found, 555.2018.

******

**Figure S25.** HPLC chromatogram of compound **3c**, C_34_H_26_N_4_O_4_

*3-(((4-((4-(4-chlorophenyl)pyrimidin-2-yl)amino)phenyl)amino)methyl) quinolin-2(1H)-one* (**4a**). Yellow solid. mp: 261-262 °C, ^1^H NMR (400 MHz, DMSO-*d_6_*) δ 4.21 (s, 2H, CH_2_), 5.37 (s, 1H, CH_2_-NH), 6.66 (d, J = 8.8 Hz, 2H, Ar-H), 7.12 (t, J = 8.0 Hz, 1H, Quin-H), 7.16 (d, J = 5.1 Hz, 1H, CH), 7.3 (d, J = 7.7 Hz, 1H, Quin-H), 7.41 (t, J = 8.3 Hz, 1H, Quin-H), 7.44 (d, J = 8.8 Hz, 2H, Ar-H), 7.51 (d, J = 8.6 Hz, 2H, Ar-H), 7.54 (d, J = 8.1 Hz, 1H, Quin-H), 7.80 (s, 1H, Quin-H), 8.07 (d, J = 8.6 Hz, 2H, Ar-H), 8.41 (d, J = 5.1 Hz, 1H, CH), 8.59 (s, 1H, NH), 11.34 (s, 1H, NH). ESI-QTOF (positive ionization) M+H calcd. for C_26_H_20_ClN_5_O, 454.1429; found, 454.1412.

**Figure S26.** HPLC chromatogram of compound **4a**, C_26_H_20_ClN_5_O

*3-(((4-((4-(4-chlorophenyl)pyrimidin-2-yl)amino)phenyl)amino)methyl)-6-methoxyquinolin-2(1H)-one* (**4b**). Yellow solid, mp: 255-256 °C, ^1^H NMR (400 MHz, DMSO-*d_6_*) δ 3.75 (s, 3H, OCH_3_). 4.17 (d, J = 5.5 Hz, 2H, CH_2_), 5.92 (t, J = 5.5 Hz, 1H, CH_2_-NH), 6.60 (d, J = 8.6 Hz, 2H, Ar-H), 7.11 (dd, J = 8.9, 2.5 Hz, 1H, Quin-H), 7.16 (s, 1H, Quin-H), 7.34 - 7.24 (m, 2H, Quin-H, CH), 7.47 (d, J = 8.6 Hz, 2H, Ar-H), 7.59 (d, J = 8.3 Hz, 2H, Ar-H), 7.74 (s, 1H, Quin-H), 8.14 (d, J = 8.3 Hz, 2H, Ar-H), 8.45 (d, J = 5.1 Hz, 1H, CH), 9.23 (s, 1H, NH), 11.78 (s, 1H, NH). ESI-QTOF (positive ionization) M+H calcd. for C_27_H_22_ClN_5_O_2_, 484.1535; found, 484.1522.

**Figure S27.** HPLC chromatogram of compound **4b**, C_27_H_22_ClN_5_O_2_

*3-(((4-((4-(4-chlorophenyl)pyrimidin-2-yl)amino)phenyl)amino)methyl)-6,7-dimethoxyquinolin-2(1H)-one* (**4c**). Yellow solid, mp: 261-262 °C, ^1^H NMR (400 MHz, DMSO-*d_6_*) δ 3.73 (s, 3H, OCH_3_), 3.78 (s, 3H, OCH_3_), 4.10 (d, J = 5.8 Hz, 2H, CH_2_), 5.84 (t, J = 5.8 Hz, 1H, CH_2_-NH), 6.56 (d, J = 8.9 Hz, 2H, Ar-H), 6.86 (s, 1H, Quin-H), 7.12 (s, 1H, Quin-H), 7.24 (d, J = 5.2 Hz, 1H, CH), 7.43 (d, J = 8.8 Hz, 2H, Ar-H), 7.56 (d, J = 8.6 Hz, 2H, Ar-H), 7.66 (s, 1H, Quin-H), 8.11 (d, J = 8.6 Hz, 2H, Ar-H), 8.42 (d, J = 5.2 Hz, 1H, CH), 9.19 (s, 1H, NH), 11.63 (s, 1H, NH). ESI-QTOF (positive ionization) M+H calcd. for C_28_H_24_ClN_5_O_3_, 514.1646; found, 514.1618.

**Figure S28.** HPLC chromatogram of compound **4c**, C_28_H_24_ClN_5_O_3_

*3-(((4-((4-(naphthalen-2-yl)pyrimidin-2-yl)amino)phenyl)amino) methyl) quinolin-2(1H)-one* (**5a**). Yellow solid, mp: 294-295 °C, ^1^H NMR (400 MHz, DMSO-*d_6_*) δ 4.20 (d, J = 5.6 Hz, 2H, CH_2_), 5.90 (t, J = 5.9 Hz, 1H, CH_2_-NH), 6.65 (d, J = 8.6 Hz, 2H, Ar-H ), 7.15 (t, J = 7.5 Hz, 1H, Quin-H), 7.35 (d, J = 8.2 Hz, 1H, Quin-H), 7.42 (d, J = 5.1 Hz, 1H, CH), 7.47 (t, J = 7.6 Hz, 1H, Quin-H), 7.53 (d, J = 8.6 Hz, 2H, Ar-H), 7.67 - 7.56 (m, 3H, Ar-H), 7.81 (s, 1H, Ar-H), 7.99 (d, J = 8.3 Hz, 1H, Ar-H), 8.05 (d, J = 8.7 Hz, 2H, Ar-H), 8.26 (d, J = 8.5 Hz, 1H, Quin-H), 8.49 (d, J = 5.1 Hz, 1H, CH), 8.72 (s, 1H, Quin-H), 9.25 (s, 1H, NH), 11.89 (s, 1H, NH). ESI-QTOF (positive ionization) M+H calcd. for C_30_H_23_N_5_O, 470.1975; found, 470.1968.

**Figure S29.** HPLC chromatogram of compound **5a**, C_30_H_23_N_5_O

*6-methoxy-3-(((4-((4-(naphthalen-2-yl)pyrimidin-2-yl)amino)phenyl) amino)methyl)quinolin-2(1H)-one* (**5b**). Yellow solid, mp: 294-295 °C, ^1^H NMR (400 MHz, DMSO-*d_6_*) δ 3.75 (s, 3H, OCH_3_). 4.20 (d, J = 5.4 Hz, 2H, CH_2_), 5.93 (t, J = 5.4 Hz, 1H, CH_2_-NH), 6.64 (d, J = 8.7 Hz, 2H, Ar-H), 7.11 (dd, J = 8.9, 2.3 Hz, 1H, Quin-H), 7.17 (s, 1H, Quin-H), 7.29 (d, J = 8.9 Hz, 1H, Quin-H), 7.42 (d, J = 5.1 Hz, 1H, CH), 7.53 (d, J = 8.5 Hz, 2H, Ar-H), 7.67 - 7.56 (m, 2H, Ar-H), 7.77 (s, 1H, Ar-H), 7.98 (d, J = 7.4 Hz, 1H, Ar-H), 8.04 (d, J = 8.7 Hz, 2H, Ar-H), 8.25 (d, J = 8.5 Hz, 1H, Ar-H), 8.49 (d, J = 5.1 Hz, 1H, CH), 8.72 (s, 1H Quin-H), 9.26 (s, 1H, NH), 11.80 (s, 1H, NH). ESI-QTOF (positive ionization) M+H calcd. for C_31_H_25_N_5_O_2,_ 500.2081; found, 500.2065.

**Figure S30.** HPLC chromatogram of compound **5b**, C_31_H_25_N_5_O_2_

*6,7-dimethoxy-3-(((4-((4-(naphthalen-2-yl)pyrimidin-2-yl)amino)phenyl )amino)methyl)quinolin-2(1H)-one* (**5c**). Yellow solid, mp: 266-267 °C, ^1^H NMR (400 MHz, DMSO-*d_6_*) δ 3.72 (s, 3H, OCH_3_). 3.79 (s, 3H, OCH_3_), 4.13 (d, J = 5.5 Hz, 2H, CH_2_), 5.84 (t, J = 6.1 Hz, 1H, CH_2_-NH), 6.59 (d, J = 8.8 Hz, 2H, Ar-H), 6.86 (s, 1H, Quin-H), 7.12 (s, 1H, Quin-H), 7.39 (d, J = 5.2 Hz, 1H, CH), 7.48 (d, J = 8.8 Hz, 2H, Ar-H), 7.62 - 7.52 (m, 2H, Ar-H), 7.68 (s, 1H, Quin-H), 8.10 - 7.88 (m, 3H, Ar-H), 8.23 (dd, J = 8.6, 1.7 Hz, 1H, Ar-H), 8.46 (d, J = 5.2 Hz, 1H, CH), 8.69 (s, 1H, CH, Ar-H), 9.21 (s, 1H, NH), 11.64 (s, 1H, NH). ESI-QTOF (positive ionization) M+H calcd. for C_32_H_27_N_5_O_3_, 530.2187 found, 530.2169.

**Figure S31.** HPLC chromatogram of compound **5c**, C_32_H_27_N_5_O_3_

*3-((4-(4-(4-chlorophenyl)pyrimidin-2-yl)piperazin-1-yl)methyl)quinolin-2(1H)-one* (**6a**). White solid, mp: 251-252 °C, ^1^H NMR (400 MHz, DMSO-*d_6_*) δ 2.58 (br, 4H, CH_2_ x 2). 3.49 (s, 2H, CH_2_), 3.90 (br, 4H, CH_2_ x 2), 7.19 (t, J = 8.1 Hz, 1H, Quin-H), 7.23 (d, J = 5.2 Hz, 1H, CH), 7.33 (d, J = 8.1 Hz, 1H, Quin-H), 7.48 (t, J = 8.5 Hz, 1H, Quin-H), 7.58 (d, J = 8.6 Hz, 2H, Ar-H), 7.71 (d, J = 7.1 Hz, 1H, CH), 7.94 (s, 1H, Quin-H), 8.17 (d, J = 8.6 Hz, 2H, Ar-H), 8.46 (d, J = 5.1 Hz, 1H, CH), 11.81 (s, 1H, NH). ESI-QTOF (positive ionization) M+H calcd. for C_24_H_22_ClN_5_O, 432.1586; found, 432.1585.

**Figure S32.** HPLC chromatogram of compound **6a**, C_24_H_22_ClN_5_O

*3-((4-(4-(4-chlorophenyl)pyrimidin-2-yl)piperazin-1-yl)methyl)-6-methoxyquinolin-2(1H)-one* (**6b**). White solid, mp: 262-263 °C, ^1^H NMR (400 MHz, DMSO-*d_6_*) δ 2.58 (br. 4H, CH_2_ x 2), 3.48 (s, 2H, CH_2_), 3.80 (s, 3H, OCH_3_), 3.90 (br, 4H, CH_2_ x 2), 7.13 (d, J = 8.8 Hz, 1H, Quin-H), 7.22 (d, J = 5.0 Hz, 1H, CH), 7.26 (d, J = 8.8 Hz, 2H, Quin-H), 7.27 (s, 1H, Quin-H), 7.57 (d, J = 8.2 Hz, 2H, Ar-H), 7.90 (s, 1H Quin-H), 8.16 (d, J = 8.2 Hz, 2H, Ar-H), 8.46 (d, J = 5.0 Hz, 1H, CH), 11.70 (s, 1H, NH). ESI-QTOF (positive ionization) M+H calcd. for C_25_H_24_ClN_5_O_2_, 462.1691; found, 462.1687.

**Figure S33.** HPLC chromatogram of compound **6b**, C_25_H_24_ClN_5_O_2_

*3-((4-(4-(4-chlorophenyl)pyrimidin-2-yl)piperazin-1-yl)methyl)-6,7-dimethoxyquinolin-2(1H)-one* (**6c**). White solid, mp: 231-231°C, ^1^H NMR (400 MHz, CDCl_3_) δ 2.71 (br, 4H, CH_2_). 3.68 (s, 2H, CH_2_), 6.87 (s, 1H), 394 (br, 4H, CH_2_), 3.98 (s, 6H, OCH_3_), 6.89 (d, J = 5.0 Hz, 1H, CH), 6.90 (s, 1H, CH, Quin-H), 6.98 (s, 1H, Quin-H), 7.42 (d, J = 8.2 Hz, 2H, H-Ar), 7.86 (s, 1H, Quin-H), 7.97 (d, J = 8.2 Hz, 2H, H-Ar), 8.37 (d, J = 5.0 Hz, 1H, CH), 12.00 (s, 1H, NH). ESI-QTOF (positive ionization) M+H calcd. for C_26_H_26_ClN_5_O_3_, 492.1797; found, 492.1793.

**Figure S34.** HPLC chromatogram of compound **6c**, C_26_H_26_ClN_5_O_3_

*3-((4-(4-(naphthalen-2-yl)pyrimidin-2-yl)piperazin-1-yl)methyl)quinolin-2(1H)-one* (**7a**). White solid, mp: 251-252 °C, ^1^H NMR (400 MHz, DMSO-*d_6_*) δ 2.65 (t, J = 5.1 Hz, 4H, CH_2_ x 2), 3.55 (s, 2H, CH_2_), 3.94 (t, J = 5.1 Hz, 4H, CH_2_ x 2), 7.14 (t, J = 8.5 Hz, 1H, Quin-H), 7.25 (d, J = 5.1 Hz, 1H, CH), 7.34 (d, J = 8.4 Hz, 1H, Quin-H), 7.43 (t, J = 8.4 Hz, 1H, Ar-H), 7.59 - 7.50 (m, 2H, Ar-H), 7.63 (dd, J = 7.8, 1.0 Hz, 1H, Ar-H), 7.89 (s, 1H, Ar-H), 7.96 - 7.90 (m, 1H, Ar-H), 7.99 (d, J = 8.6 Hz, 1H, Quin-H), 8.19 (dd, J = 8.5, 1.8 Hz, 1H, Quin-H), 8.45 (d, J = 5.1 Hz, 1H, CH), 8.63 (s, 1H, Quin-H), 11.24 (s, 1H, NH). ESI-QTOF (positive ionization) M+H calcd. for C_28_H_25_N_5_O, 448.2132; found, 448.2129.

**Figure S35.** HPLC chromatogram of compound **7a**, C_28_H_25_N_5_O

*6-methoxy-3-((4-(4-(naphthalen-2-yl)pyrimidin-2-yl)piperazin-1-yl)methyl) quinolin-2(1H)-one* (**7b**). White solid, mp: 247-248 °C, ^1^H NMR (400 MHz, DMSO-*d_6_*) δ 2.61 (br, 4H, CH_2_ x 2). 3.50 (s, 2H, CH_2_), 3.81 (s, 3H, OCH_3_), 3.96 (br, 4H, CH_2_ x 2), 7.13 (d, J = 8.9 Hz, 1H, Ar-H), 7.27 (d, J = 9.2 Hz, 1H, Ar-H), 7.29 (s, 1H, Quin-H), 7.38 (d, J = 5.1 Hz, 1H, CH), 7.64 - 7.54 (m, 2H, Ar-H), 7.92 (s, 1H, Quin-H), 8.01 - 7.96 (m, 1H, Ar-H), 8.04 (d, J = 8.6 Hz, 1H, Quin-H), 8.09 (d, J = 5.9 Hz, 1H, Ar-H), 8.28 (d, J = 8.6 Hz, 1H, Quin-H), 8.50 (d, J = 5.1 Hz, 1H, Ar-H), 8.75 (s, 1H, Ar-H), 11.71 (s, 1H, NH). ESI-QTOF (positive ionization) M+H calcd. for C_29_H_27_N_5_O_2_, 478.2238; found, 478.2233.

**Figure S36.** HPLC chromatogram of compound **7b**, C_29_H_27_N_5_O_2_

*6,7-dimethoxy-3-((4-(4-(naphthalen-2-yl)pyrimidin-2-yl)piperazin-1-yl)methyl)quinolin-2(1H)-one* (**7c**). White solid, mp: 262-263 °C, ^1^H NMR (400 MHz, CDCl_3_) δ 2.63 - 2.80 (m, 4H, CH_2_ x 2), 3.70 (s, 2H, CH_2_), 3.93 (s, 3H, OCH_3_), 3.99 (s, 3H, OCH_3_), 4.12 - 4.01 (m, 4H, CH_2_ x 2), 6.89 (s, 1H, CH), 6.98 (s, 1H, Quin-H), 7.08 (d, J = 5.2 Hz, 1H, Quin-H), 7.59 - 7.46 (m, 2H, CH), 8.00 - 7.81 (m, 4H), 8.15 (dd, J = 8.6, 1.7 Hz, 1H), 8.41 (d, J = 5.2 Hz, 1H, CH), 8.52 (s, 1H, Quin-H), 12.19 (s, 1H, NH). ESI-QTOF (positive ionization) M+H. calcd. for C_30_H_29_N_5_O_3_, 508.2343; found, 508.2339.

**Figure S37.** HPLC chromatogram of compound **7c**, C_30_H_29_N_5_O_3_

**References**

1 Laali, K. K., Insuasty, D., Abonia, R., Insuasty, B. & Bunge, S. D. Novel quinoline–imidazolium adducts via the reaction of 2-oxoquinoline-3-carbaldehyde and quinoline-3-carbaldehydes with 1-butyl-3-methylimidazolium chloride [BMIM][Cl]. *Tetrahedron Lett.* **55**, 4395-4399 (2014).

2 Vettorazzi, M. *et al.* Design of new quinolin-2-one-pyrimidine hybrids as sphingosine kinases inhibitors. *Bioorg. Chem.* **94**, 103414 (2020).
